# A Comprehensive Proteome and Acetyl-Proteome Atlas Reveals Molecular Mechanisms Adapting to the Physiological Changes From Pre-laying to Peak-Laying Stage in Liver of Hens (*Gallus gallus*)

**DOI:** 10.3389/fvets.2021.700669

**Published:** 2021-10-21

**Authors:** Zhang Wang, Dandan Wang, Keren Jiang, Yulong Guo, Zhuanjian Li, Ruirui Jiang, Ruili Han, Guoxi Li, Yadong Tian, Hong Li, Xiangtao Kang, Xiaojun Liu

**Affiliations:** ^1^College of Animal Science and Technology, Henan Agricultural University, Zhengzhou, China; ^2^Henan Key Laboratory for Innovation and Utilization of Chicken Germplasm Resources, Zhengzhou, China; ^3^International Joint Research Laboratory for Poultry Breeding of Henan, Zhengzhou, China

**Keywords:** liver, hens, post-translational modifications, acetylation, lipid metabolism

## Abstract

Along with sexual maturity, the liver undergoes numerous metabolic processes to adapt the physiological changes associated with egg-laying in hens. However, mechanisms regulating the processes were unclear. In this study, comparative hepatic proteome and acetyl-proteome between pre- and peak-laying hens were performed. The results showed that the upregulated proteins were mainly related to lipid and protein biosynthesis, while the downregulated proteins were mainly involved in pyruvate metabolism and were capable of inhibiting gluconeogenesis and lactate synthesis in peak-laying hens compared with that in pre-laying hens. With unchanged expression level, the significant acetylated proteins were largely functioned on activation of polyunsaturated fatty acid oxidation in peroxisome, while the significant deacetylated proteins were principally used to elevate medium and short fatty acid oxidation in mitochondria and oxidative phosphorylation. Most of the proteins which involved in gluconeogenesis, lipid transport, and detoxification were influenced by both protein expression and acetylation. Taken overall, a novel mechanism wherein an alternate source of acetyl coenzyme A was produced by activation of FA oxidation and pyruvate metabolism to meet the increased energy demand and lipid synthesis in liver of laying hens was uncovered. This study provides new insights into molecular mechanism of adaptation to physiological changes in liver of laying hens.

## Introduction

It is well-known that liver, as a central metabolic organ, acts as a critical hub for numerous physiological processes including metabolisms of glucose, lipid and cholesterol, protein, and amino acid and so forth in mammals (1, 2). In recent years, advances in mass spectrometry-based proteomics have not only provided new insights into the dynamic functional changes during liver development (3–5) and explored new roles of liver in regulating physiological processes, such as the glycine-serine-threonine metabolic axis in longevity (6) but also revealed molecular mechanisms associated with lipid metabolic diseases, such as fatty liver (7), obesity (8) and hepatocellular carcinoma (5).

The post-translational modifications (PTMs) have been implicated in the regulation of various regulatory and metabolic processes. Among them, lysine (K) acetylation (Kac) modification is an evolutionarily and highly conserved PTM mechanism and the one of the most studied PTMs to date. A large body of evidence has proved that protein acetylation has a broad regulatory effect on cellular metabolism through altering protein stability, catalytic activity, subcellular localization of metabolic enzymes, and protein–protein interactions (9, 10). Human-related studies have indicated that protein Kac modification affects cellular nuclear gene transcription and processing (11, 12), protein accumulation or folding (13, 14), DNA damage repair (15) and autophagy (16) through the regulation of enzyme activity (17), protein degradation (18), protein interactions (19), subcellular localization (20), and DNA binding ability (21), thus regulating energy metabolism and other biological processes. Specially, in terms of liver lipid metabolism, it has been proven that Kac is involved in most metabolic pathways by regulating carbon metabolism (22). Acetylated lysine residue in sterol regulatory element binding 1a (Srebp1a) stabilizes the protein by inhibiting ubiquitination (23). Deacetylation of a transactivation domain of X-box-binding protein 1 (Xbp1s) at K257 and K297 via sirtuin (sirt) 6 promotes Xbp1s protein degradation through the ubiquitin-proteasome system, which confers resistance to endoplasmic reticulum (ER) stress-induced hepatic steatosis (24). Deacetylated long-chain acyl-CoA dehydrogenase (Lcad) can enhance mitochondrial energetics, which contributes to the amelioration of lipotoxicity in hepatocytes (25). Reduced acetylation of Rptor, which is a component of the mechanistic target of rapamycin kinase (Mtor) complex 1 and a key regulator of autophagy, protects mice from hepatic steatosis induced by starvation or high-fat diet (HFD) via induction of lipophagy (26). In addition, protein acetylation in mitochondria associates with important biological pathways involved in the regulation of the pathogenesis of fatty liver disease in dairy cows (27). These studies suggest that non-histone acetylation plays an important role in the regulation of liver lipid metabolism.

In chicken, the liver is the central metabolic organ in controlling metabolic homeostasis, acting as the primary site for lipid metabolism, where more than 90% of *de novo* fatty acids were synthesized in chicken (28, 29). The synthesized lipid was packaged as lipoproteins, mainly very low-density lipoprotein (VLDL) and vitellogenin (Vtg), secreted into the blood stream and taken up by the growing oocytes via endocytotic processes involving specific cell-surface receptors (30, 31). With sex maturation, hepatic lipid metabolism is strongly activated in liver of hen. Comparative transcriptome analysis suggested that differentially expressed genes (DEGs) were significantly enriched in oxidation reduction, sterol and cholesterol metabolic processes, and lipid biosynthetic processes between the liver of peak-laying hens at 30 weeks old (30 w) and the liver of pre-laying hens at 20 w (32). Integrative analysis of transcriptomic data related to the liver of laying hens further revealed the important roles of liver in lipid metabolism and linked it with egg yolk formation and egg fertilization as well (33–35). However, the roles of lysine acetylation modification of proteins in liver lipid metabolism in chicken are rarely reported until now.

To elucidate the molecular mechanisms of lysine acetylation modification in chicken liver for adapting to the physiological changes of laying hens, the proteome and acetyl-proteome of livers in pre-laying hens (20 w) and peak-laying hens (30 w) were investigated using tandem mass tags (TMT) labeling in combination with liquid chromatograph mass spectrometer (LC-MS/MS) analysis in this study. Comprehensive analyses were used to resolve the molecular mechanisms of adapting to the physiological changes from pre-laying to peak-laying stages in hens. The present results provide a novel insight into the roles of proteins and protein acetylation in liver metabolisms in laying hen.

## Materials and Methods

### Animals and Sampling

Our previous study had shown that the lipid content was significantly higher in peak-laying hens at 30 w than that in pre-laying hens at 20 w (36). In this study, a total of 80 healthy Lushi blue-shelled-egg (LS) chickens, hatched in the same batch and possessing similar body weight, were housed in separate cages and raised in the same environmental conditions with food and water *ad libitum* at the Animal Center of Henan Agricultural University. When they reached 20 and 30 weeks of age, three birds were randomly selected and slaughtered, respectively. The liver tissue (designated as L20w1, L20w2, and L20w3 in the pre-laying group, and L30w1, L30w2, and L30w3 in the peak-laying group, respectively), from the same position of right lobe of the liver in each bird was immediately dissected, snap-frozen in liquid nitrogen, then stored at −80°C until use.

### Protein Extraction and Enzymolysis

The total protein was extracted from liver tissues by using radio immunoprecipitation assay (RIPA) lysis buffer including 10 mM Tris-HCl, 1% Triton X-100, 1% sodium deoxycholate, and 0.1% sodium dodecyl sulfate (SDS). The protein concentration was assessed by bicinchoninic acid (BCA) assay kit (Applygen, Beijing, China) according to the manufacturer's protocol. Two hundred micrograms of protein were divided into two equal parts for each of samples, which were used for quantification of proteome and acetyl-proteome, respectively.

For digestion, the protein was reduced with 5 mM dithiothreitol (Sigma Alrdich, St. Louis, MO, USA) and incubated for 30 min at 56°C, and subsequently alkylated with 55 mM iodoacetamide (Sigma Alrdich) for 15 min at room temperature in darkness. After being washed with 100 mM TEAB buffer (Sigma Alrdich) two times, trypsin (Promega, Beijing, China) was added with a ratio of 1:50 (trypsin:protein) and incubated overnight at room temperature for the first digestion, then added with a ratio of 1:100 (trypsin:protein) and incubated for a further 4 h for the second digestion.

### Western Blotting

Total protein was extracted and its concentration was assessed by using the BCA protein quantification kit (Applygen, Beijing, China). The total protein (50 μg) was separated on a 10% SDS-PAGE gel and transferred to methanol-activated polyvinylidene difluoride membranes (Millipore, Billerica, MA, USA). The membranes were blocked in 5% bovine serum albumin blocking solution for 1 h at room temperature and incubated with primary pan-acetyl-lysine antibody (PTM-101; 1:1000 dilution), primary pan-succinyl-lysine antibody (PTM-419; 1:1000 dilution), and primary pan-malonyl-lysine antibody (PTM-902; 1:6400 dilution; Jingjie Biotechnology Co, Hangzhou, China) overnight at 4°C, respectively. Subsequently, the membranes were washed with PBS-T and incubated with peroxidase conjugated secondary antibodies goat anti-mouse IgG (1:10,000 dilution; Thermo Fisher Scientific, Waltham, MA, USA) for 1 h at room temperature. Protein bands were visualized using an enhanced chemiluminescence plus system (GE Healthcare, Marlborough, MA, USA), and optical density of the bands were analyzed by AlphaView 3.0 (Alpha Innotech, San Jose, CA, USA).

### TMT Labeling and HPLC Fractionation

The trypsinized peptides were desalted with Strata X C18 (Phenomenex, Torrance, CA, USA) and vacuum freeze-dried. The desalted peptides were then solubilized with 0.5 M TEAB and labeled using 6-plex TMT reagents (Thermo Fisher Scientific) for separation by high-performance liquid chromatography (HPLC) according to the manufacturer's instructions. In brief, each TMT was dissolved with acetonitrile, and the mixed regent was added to each of the extracted peptide according to the scheme reported in Additional File 1. After incubation at room temperature for 2 h, the reaction was quenched and subsequently the labeled samples were mixed. Finally, the labeled peptides were desalted and then vacuum freeze-dried. The peptide extracts were then subjected to C18 column (Agilent, Santa Clara, CA, USA) to remove unreacted compounds and salts before strong cation exchange fractionation. The peptide gradient was set as follows: 8–32% acetonitrile, pH = 9. The peptides then were separated into 60 components in 60 min. Finally, the peptides were combined into four fractions, which were vacuum freeze-dried and stored for LC-MS/MS analysis.

### Acetylation Modification Enrichment Assay

To obtain acetylated peptide segments, the peptides were dissolved in IP buffer (100 mM NaCl, 1 mM EDTA, 50 mM Tris-HCl, 0.5% NP-40, pH 8.0) and the supernatant was transferred to pre-washed anti-acetylated resin (Jingjie Biotechnology Co, Hangzhou, China) and gently shaken overnight at 4°C. After the incubation, the resin was washed four times with IP buffer followed twice with deionized water. Finally, the resin was eluted with 0.1% trifluoroacetic acid three times to remove the peptides bound to the resin. The elute was collected and dried by vacuum freezing. After drying, the peptide was desalted according to the instructions of ZipTips C18 (Sigma, Shanghai, China), then dried by vacuum freezing for LC-MS/MS analysis.

### LC-MS/MS Analysis

Enzymatic peptides enriched from acetylated antibodies were used for quantitative analysis of protein acetylation modifications; otherwise, they were used for protein quantification by LC-MS/MS. Samples were analyzed with a Q-Exactive Plus mass spectrometer, coupled to an EASY-nLC 1000 ultra-high performance liquid chromatography system (Thermo Fisher Scientific). The peptides were separated using mobile phases, water A and B, both containing 0.1% formic acid (v/v). The liquid phase gradients were set as follows: 0–19 min, 8–20% B; 19–32 min, 20–32% B; 32–36 min, 32–80% B; 36–40 min, 80% B. The flow rate was maintained at 500 nl/min.

The peptides were injected into the NSI ion source with 2.0 kV of electrospray voltage. The *m/z* scan range was 350–1,800 for a full scan, and intact peptides were detected in the OrbitraP at a resolution of 70,000. Peptides were then selected for MS/MS using the NCE setting as 28, and the fragments were detected in the OrbitraP at a resolution of 17,500. A data-dependent procedure that alternated between one MS scan followed by 20 MS/MS scans with 15.0 s dynamic exclusion was followed. Automatic gain control was set at 5E4. The fixed first mass was set as 100 *m/z*.

### Database Search

Secondary mass spectrometry data were searched using Maxquant (v1.5.2.8) in the Gallus_gallus_uniprot_9031 database (https://www.uniprot.org/ proteomes/ ?query = taxonomy : 9031), which was from anti-acetylated resin peptides or not, respectively. An anti-library was added to calculate the false-positive rate (FDR) caused by random matching. The common contamination libraries were added to eliminate the effect of contaminating proteins. The search parameters were set as follows: Trypsin/P for the enzymatic cutting mode, two for the number of missed cut sites; the minimum length of the peptide segment was set to seven amino acid residues; the maximum modification number of the peptide segment was set to five; the mass error tolerance of the primary parent ions of the First search and Main search was set to 20 and 5 ppm, respectively, and the mass error tolerance of the secondary fragment ions was 0.02 Da. The cysteine alkylation was set to fixed modification, and the variable modifications were oxidation of methionine, acetylation of protein N-terminal, deamidation, and acetylation of lysine. The quantitative method was set to TMT-6 plex, and the FDR of protein identification and the peptide-spectrum match identification was set to 1%. Acetylation levels were normalized by protein abundance.

After normalization, the abundance matrix file from proteins and acetylated lysine sites in each sample were used as the input data to perform principal component analysis (PCA), which was maximized in a plane scatter plot *via* TBtools [v1.09852; (37)]. In addition, the abundance data were also as the input data to calculate the Euclidean distance between samples and call the hcluster function to perform hierarchical clustering analysis in R platform.

### Motif Analysis

The Motif-x software (V5.0.2) was used to analyze the motif features of the modified sites. Comparative analysis of a modified 21-mer sequence model consisting of all identified modification sites (10 sites upstream and 10 sites downstream) was performed. When the number of peptides in a certain characteristic sequence was >20 and the statistical test *p* < 0.01, it was considered as a motif of the modified peptides. The NetSurfP-2.0 software (https://services.healthtech.dtu.dk/service.php?NetSurfP-2.0) was used to analyze the locations of acetylated and/or non-acetylated lysine in the secondary structures of proteins.

### Annotation and Functional Enrichment Analysis of Proteins

Gene ontology (GO) annotation at the proteomic level was performed with the UniProt-GOA database (http://www.ebi.ac.uk/GOA/). For the biological pathways annotation of proteins, the proteins were firstly annotated using the Kyoto Encyclopedia of Genes and Genomes (KEGG) online service tool KAAS (v2.0; https://www.genome.jp/tools/kaas/), and the annotated proteins were then matched to the corresponding pathways in the database via KEGG mapper (v2.5; http://www.kegg.jp/kegg/mapper.html). For the subcellular localization annotation of proteins, the Wolfpsor (v0.2; http://www.genscript.com/psort/wolf_psort.html) was used, and only ratios with *p* ≤ 0.05 were considered significant when GO and KEGG enrichment tests were performed.

The differentially acetylated proteins were divided into four categories according to differentially expressed multiples, designated as Q1–Q4: Q1 (0 < L20w/L30w Ratio ≤ 1/1.3), Q2 (1/1.3 < L20w/L30w Ratio ≤ 1/1.3), Q3 (1.3 < L20w/L30w Ratio ≤ 1.5), and Q4 (L20w/L30w Ratio > 1.5). The clustering relationships are visualized using a heat map drawn by the function heatmap.2 in the R package gplots (v.2.0.3).

### PPI Network Analysis

All protein sequences of the differently acetylated proteins were searched in STRING database (v.10.5) for protein Network. The protein–protein interactions were extracted according to the confidence score >0.7 (high confidence) and clustered and visualized using Cytoscape software (v3.8.2).

### Statistical Analysis

In this study, completely random design was performed for sampling according to random number. Each replicate was served as the experimental unit for all statistical analyses.

To identify significantly differently expressed protein (SDEP), the mean of the quantitative value of each protein in the three biological replicates was calculated then converted into log2 value to make the data conform to a normal distribution. The ratio of the average quantitative value of the same protein in 20 and 30 w birds (L30w/L20w) was determined. The two-sample two-tailed *t*-test was used to evaluate the statistical significance of expression differences of proteins between 20 and 30 w birds. The differentially expressed proteins with *p* ≤ 0.05, 1.3 ≤ L30w/L20w ratio ≤ 1/1.5 was considered to be the SDEPs. The same statistical analysis was used to examine the statistical significance of Kac sites.

For functional enrichment analysis, Fisher's exact double-ended test was used to analyze statistical significance of enriched GO terms and KEGG pathways from SDEPs based on a Perl module (https://metacpan.org/pod/Text::NSP::Measures::2D::Fisher). The same statistical analysis was used to determine the statistical significance of enriched GO terms and KEGG pathways from differentially acetylated proteins.

All statistical analyses were performed by using SPSS version 23.0 (IBM, Chicago, IL, USA). The differences with *p* ≤ 0.05 were considered to be statistically significant.

## Results

### Differentially Expressed Proteins in Livers Between Pre- and Peak-Laying Hens

**Figure 1:**
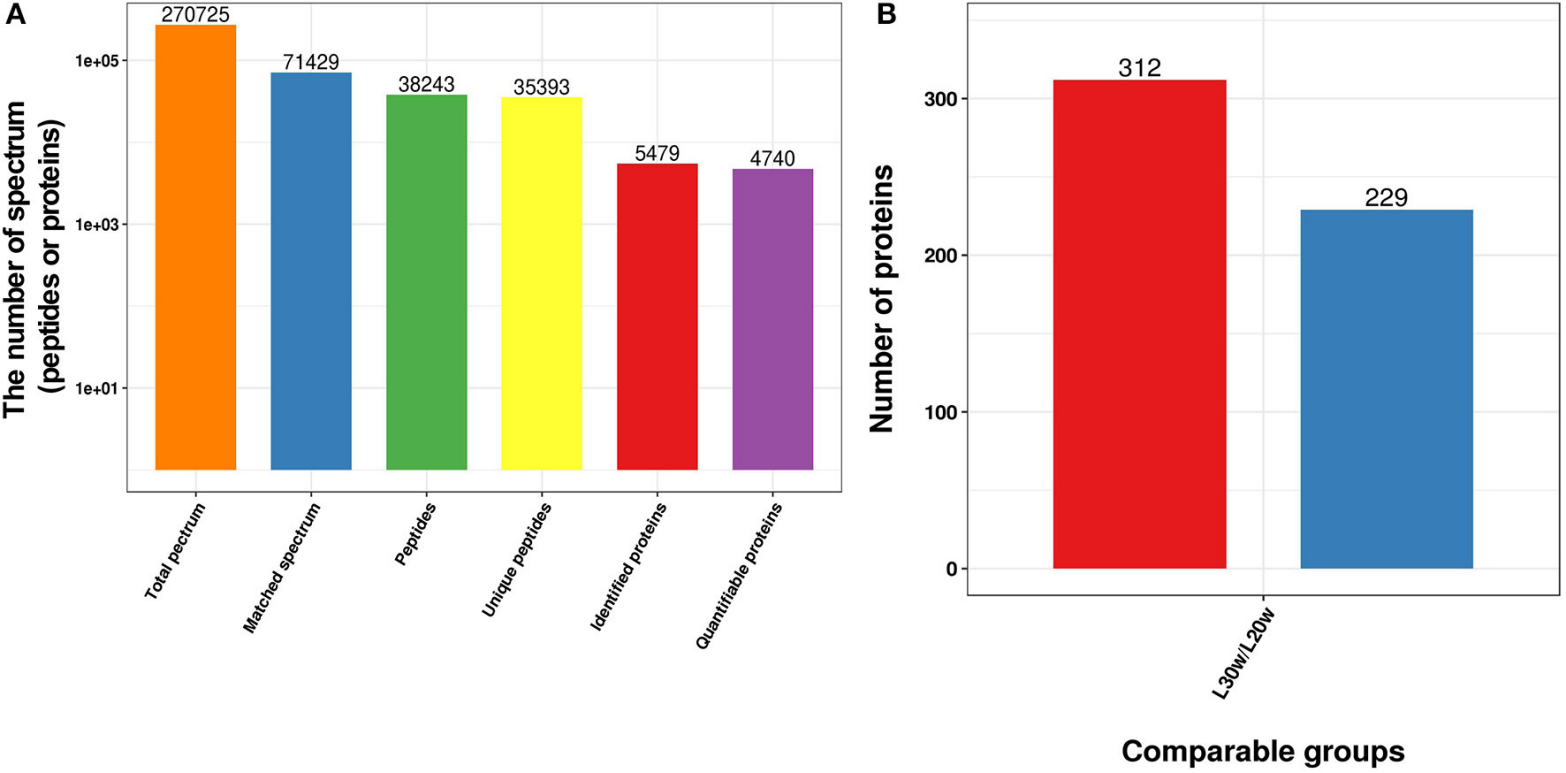
Overview of the SDEPs in livers of chickens at the age of 20 and 30 w. **(A)** Basic statistics of mass spectrometry data. **(B)** Statistics of SDEPs.

The soluble proteins of livers obtained from both pre-laying (20 w) and peak-laying (30 w) hens were separately analyzed using quantitative proteomics with 3 replications (Supplementary Figure 1A). A total of 5,479 proteins were identified, and 4,740 were quantified (Figure 1A). Of the 4,740 proteins, 541 SDEPs including 312 upregulated (*p* ≤ 0.05 and 1.3 ≤ L30w/L20w ratio) and 229 downregulated (*p* ≤ 0.05 and L30w/L20w ratio ≤ 1/1.5) were identified in livers of peak-laying hens compared with the pre-laying hens (Figure 1B and Supplementary Table 1). The PCA of the proteome profiles indicated that the first principal component clearly distinguished the two sets of samples (Supplementary Figure 1B). Pearson correlations of log2 relative quantitative value between replications were over 0.65, and relative standard deviations of protein quantification values between replicate samples were <0.1 (Supplementary Figures 1C,D). The results showed that the three biological repeats in each group were statistically consistent, indicating that the data were reliable.

### Functional Annotation of SDEPs

**Figure 2:**
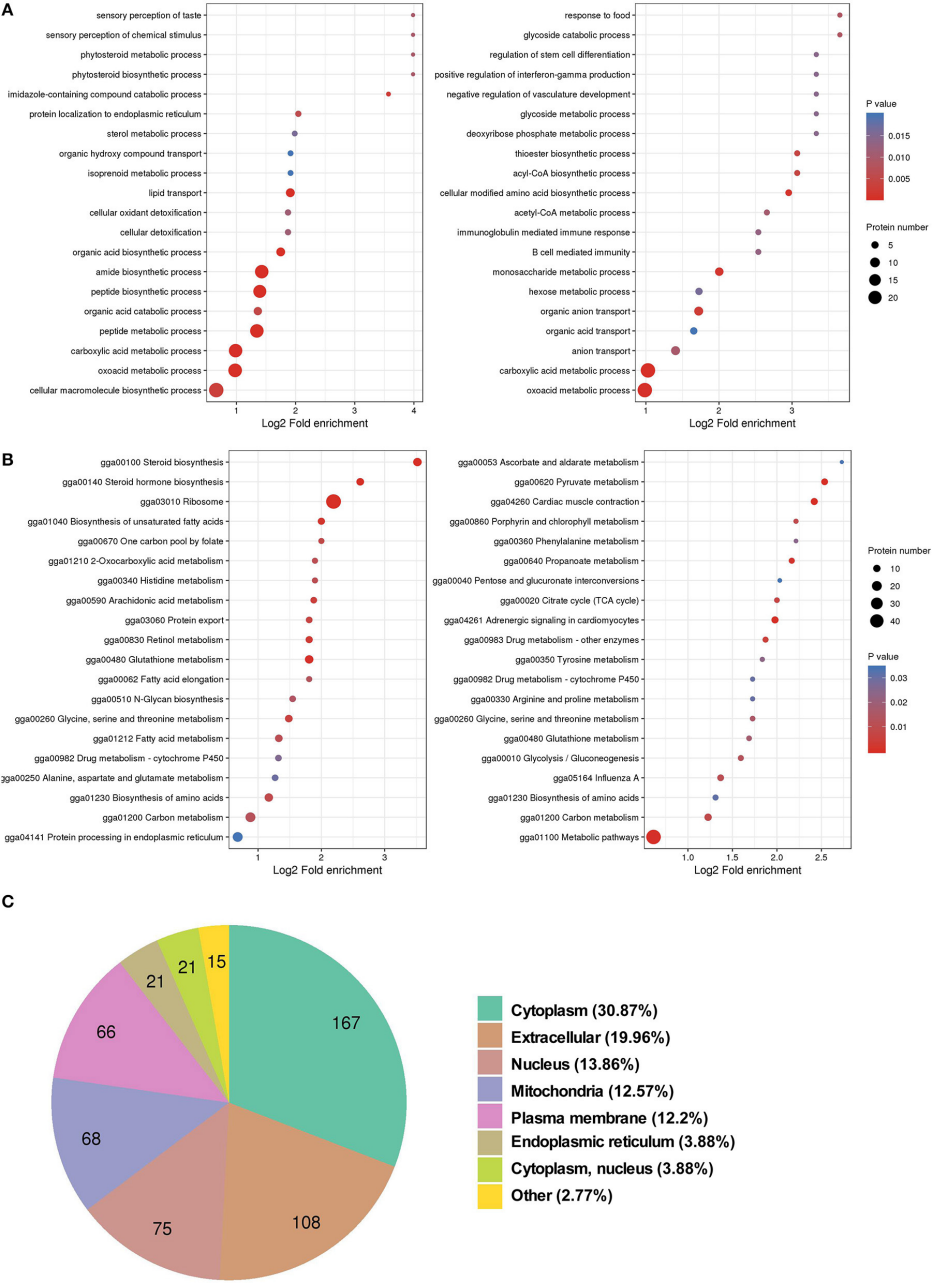
GO and KEGG enrichment analyses, and subcellular localization of SDEPs. **(A)** GO enrichment analysis of SDEPs. The significantly enriched (*p* ≤ 0.05) biological process, cellular component, and molecular function categories are shown at the left box (for upregulated proteins) and right box (for downregulated proteins), respectively. **(B)** KEGG enrichment analysis of SDEPs. The significantly enriched (*p* ≤ 0.05) pathways are shown at left box (for upregulated proteins) and right box (for downregulated proteins). **(C)** Subcellular localization of SDEPs.

To understand the biological function of SDEPs, the GO term enrichment analysis was conducted. The results showed that most of the upregulated proteins were significantly enriched in the peptide biosynthetic process (*p* < 0.01) and lipid transport (*p* < 0.01), while most of the downregulated proteins were related to the carboxylic acid metabolic process (*p* < 0.01), acetyl coenzyme A (acyl-CoA) metabolic process (*p* = 0.01), and glucuronosyltransferase activity (*p* = 0.01; Figure 2A). Further KEGG pathway analysis indicated that many upregulated proteins were significantly enriched in the pathways of ribosome (*p* < 0.01), such as most members of the ribosomal protein (Rpl) family (Supplementary Figure 2); fatty acid metabolism (*p* = 0.02), such as fatty acid desaturase (Fads) 1 and 2, acyl-CoA carboxylase (Acac), elongation of very long-chain fatty acids protein (Elovl) 2 and 17-beta-hydroxysteroid dehydrogenase enzyme (Hsd17b)12; and steroid hormone synthesis (*p* < 0.01), such as proteins cytochrome P450 (Cyp) 2d49 and Hsd17b. In addition, some upregulated proteins involved in fatty acid transport, such as proteins Vtg, heart fatty acid binding protein (Fabp3), apolipoprotein B (Apob) and microsomal triglyceride transfer protein large subunit-like protein (Mttpl); triglyceride (TG) synthesis, such as acyl-CoA synthetase long-chain family member 2 (Acsf2), glycerol-3-phosphate acyltransferase 4 (Gpat4), and 1-acylglycerol-3-phosphate O-acyltransferase 2 (Agpat2); cholesterol synthesis, such as farnesyl-diphosphate farnesyl transferase 1 (Fdft1), squalene epoxidase (Sqle), lanosterol synthase (Lss), lanosterol-14α-demethylase (Cyp51a1), 24-dehydrocholesterol reductase (Dhcr24), lamin B receptor (LBR), and emopamil binding protein (Ebp); and tricarboxylic acid (TCA) cycle, such as malate dehydrogenase 2 (Mdh2) and isocitrate dehydrogenase (NAD+; Idh) 2, suggesting an active lipid metabolism and adenosine triphosphate (ATP) synthesis in the liver of laying hens. For the downregulated SDEPs, many of them were significantly enriched in pyruvate metabolism (*p* < 0.01), including gluconeogenesis, such as phosphoenolpyruvate carboxykinase 1 (Pck1) and pyruvate carboxylase (Pc); acyl-CoA synthesis, such as pyruvate dehydrogenase kinase (Pdk4) and acyl-CoA synthetase (Acss) short-chain family member 1 (Acss1l); and lactic acid synthesis, such as L-lactate dehydrogenase A (Ldha) and Idh 3 catalytic subunit alpha (Idh3a; Figure 2B). It indicated that the downregulated proteins might inhibit the pyruvate metabolites flux to gluconeogenesis and lactic acid synthesis. However, downregulated PDK4 elevated dephosphorylation of pyruvate dehydrogenase (PDH) complex, thus promoting the conversion of pyruvate to acyl-CoA (38). In addition, subcellular localization analysis indicated that most SDEPs localized in the cytoplasm or ER, followed by nucleus and mitochondria (Figure 2C and Supplementary Table 1). In general, the liver in 30 w birds exhibited higher synthetizing ability for protein, lipid, and steroid hormone synthesis than that in 20 w birds.

### Profiling of Lysine Acetylated Proteins in Liver of Chicken

**Figure 3:**
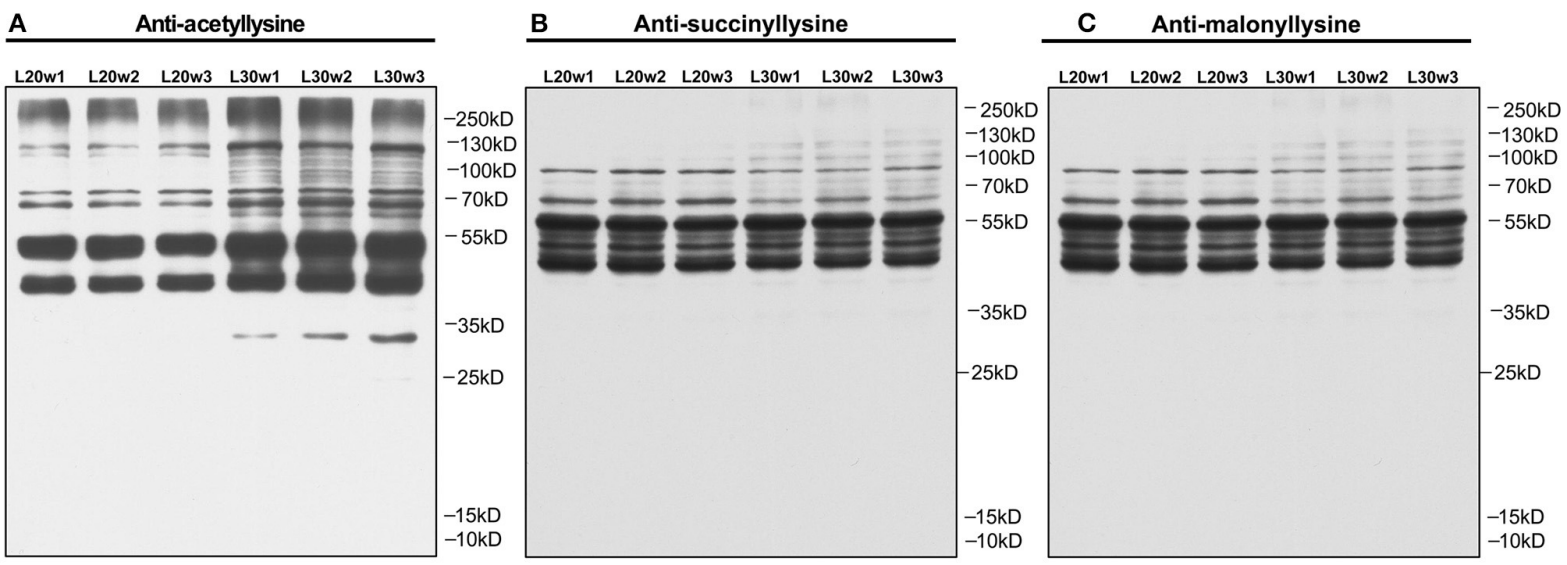
PTM analysis of liver proteins by Western blot with corresponding pan-antibodies in pre-laying (20 w) and peak-laying (30 w) hens. **(A)** Acetylation. **(B)** Succinylation. **(C)** Malonylation.

To explore the epigenetic effects on the liver metabolism in chicken, the PTMs of hepatic proteins were analyzed with pan anti-acetyl-lysine antibody, pan anti-succinyl-lysine antibody, and pan anti-malonyl-lysine antibodies by Western blot. The results showed increased protein acetylation levels in the livers of peak-laying hens comparing to pre-laying hens (Figure 3A). However, the succinylation (Figure 3B) and malonylation (Figure 3C) of hepatic proteins remained unchanged between pre-laying and peak-laying hens. Therefore, the protein acetylation was further dissected.

**Figure 4:**
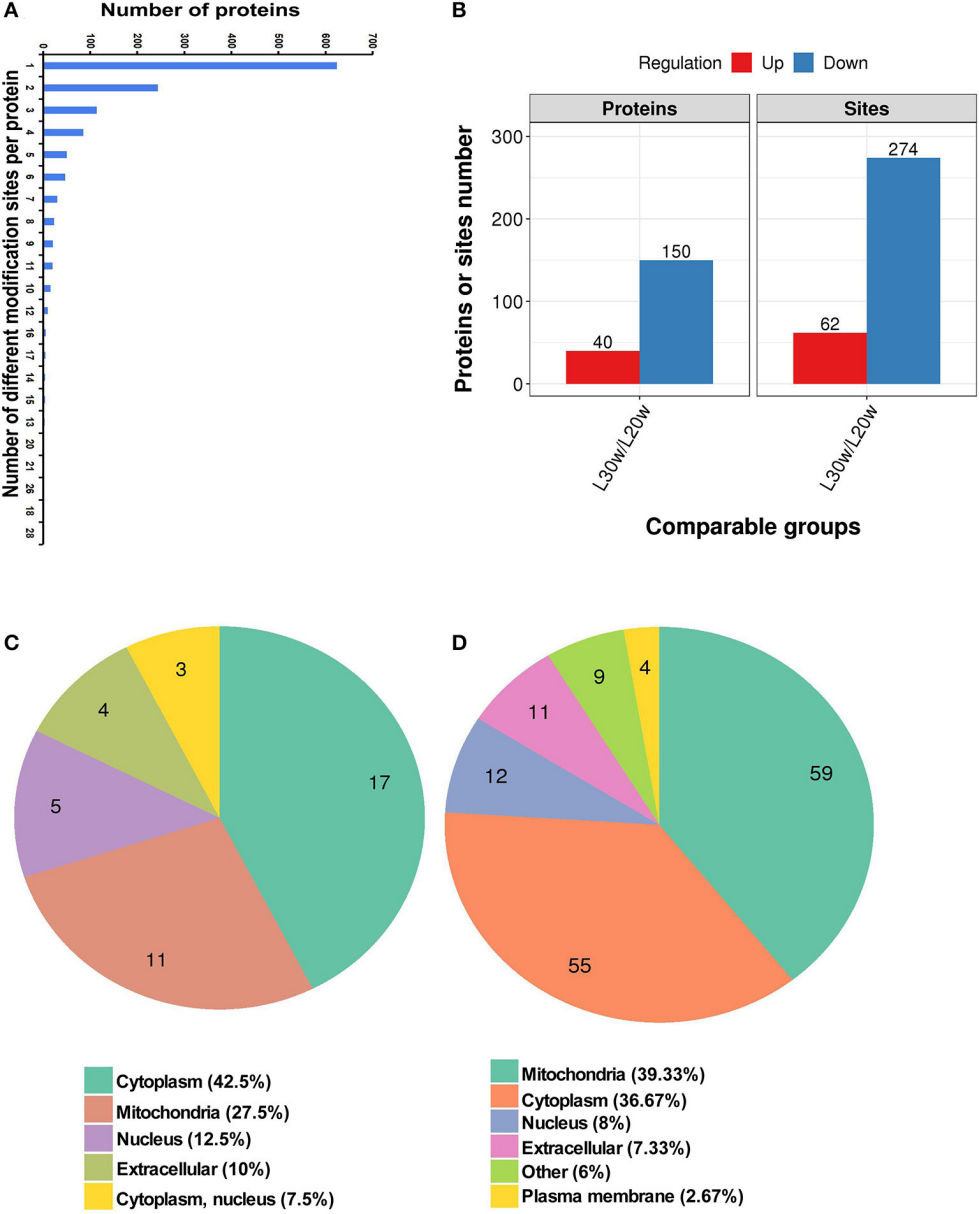
Identification and functional enrichment of lysine-acetylated proteins. **(A)** Distribution of acetylated lysine site in corresponding proteins. **(B)** Numbers of acetylated lysine site and corresponding proteins. **(C)** Subcellular localization of differentially upregulated kac proteins. **(D)** Subcellular localization of differentially downregulated kac proteins.

A total of 3,942 sites from 1,313 proteins were acetylated. Among the acetylated sites, 3,607 sites from 1,215 of proteins have been quantitated (Table 1). Six hundred twenty-four proteins were detected with only one acetylated lysine site, and 23 proteins were identified with more than 20 lysine acetylation sites (Figure 4A). Compared with that in pre-laying hens, 40 proteins with significantly upregulated acetylation levels (PwSUALs) on 62 lysine sites (*p* ≤ 0.05 and 1.3 ≤ L30w/L20w ratio) and 148 proteins with significant downregulated acetylation levels (PwSDALs) on 274 lysine sites (*p* ≤ 0.05 and L30w/L20w ratio ≤ 1/1.5) were identified in the liver of peak-laying hens (Figure 4B and Supplementary Table 2). The proteins with significantly differential acetylation levels were mainly located on mitochondria and cytoplasm (Figures 4C,D).

**Table 1:** MS/MS spectrum database search analysis summary.

**Total**	**Matched**	**Peptides**	**Modified**	**Identified**	**Quantifiable**	**Identified**	**Quantifiable**	**Normalized**	**Normalized**
**spectrum**	**spectrum**		**peptides**	**proteins**	**proteins**	**sites**	**sites**	**proteins**	**sites**
71,631	9,683 (13.5%)	4,263	3,829	1,313	1,215	3,924	3,607	790	2622

The PCA among samples indicated that the biological replicates in both groups were highly correlated (Supplementary Figure 3A). Additionally, the distribution of peptide mass errors was close to zero, and most of them were <10 ppm (Supplementary Figure 3B). Moreover, the length of the peptide segments showed a theoretical distribution (Supplementary Figure 3C). These results reflected appropriate sample preparation and data reliability.

### Sequence Preference of Acetylated Lysine Sites

**Figure 5:**
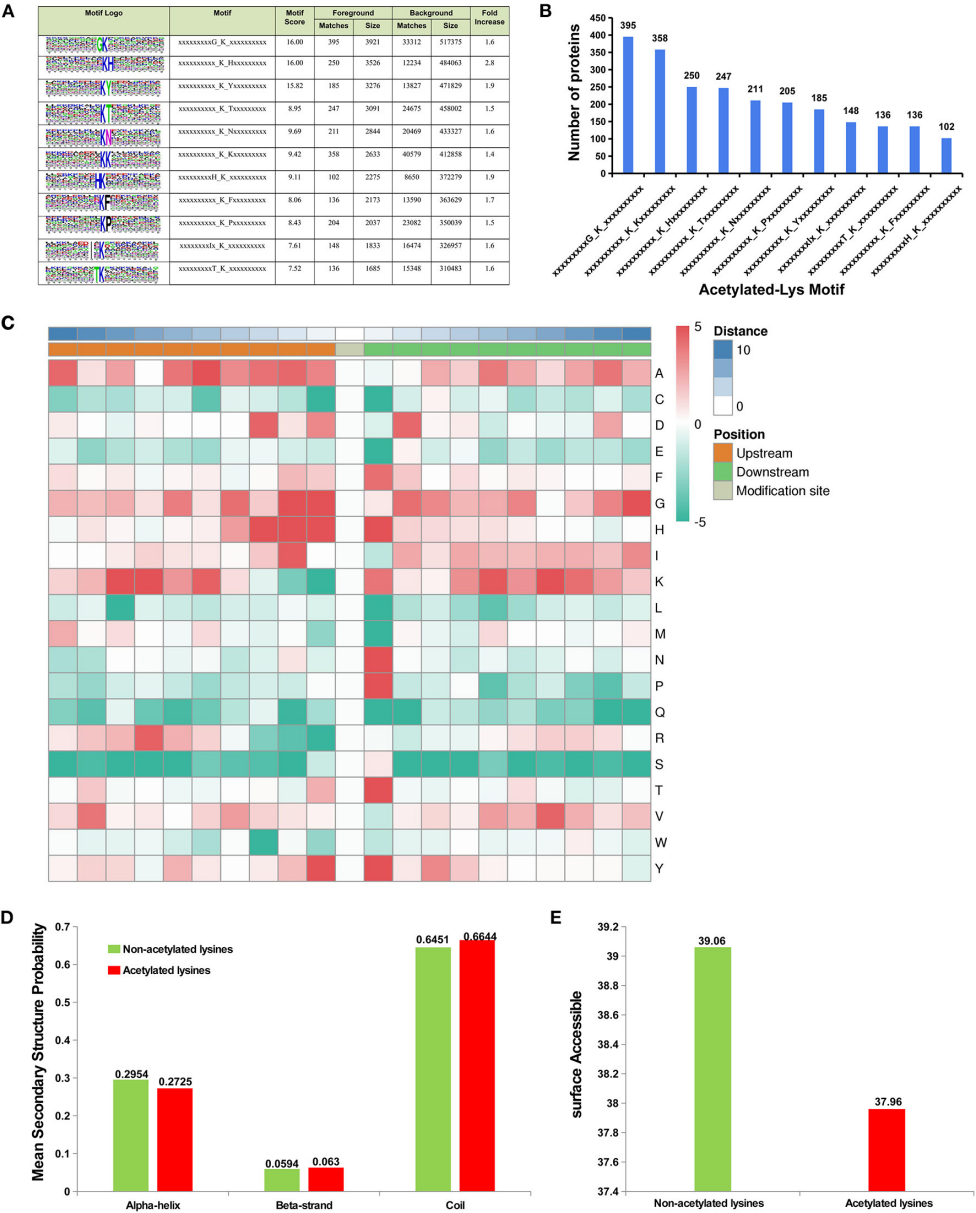
Sequence preference of lysine acetylation sites. **(A)** Probable sequence motifs of acetylation sites in liver tissue identified using Motif-X. **(B)** Number of identified peptides containing acetylated lysines and their probable motifs. **(C)** Heat map showing the relative frequencies of amino acids in specific positions, including enrichment (red) or depletion (green) of amino acids flanking the acetylated lysine in liver proteins. Location probabilities of acetylated and/or non-acetylated lysines in **(D)** protein secondary structures (alpha-helix, beta-strand, and coil) and **(E)** surface accessibility.

To analysis the sequence preference of the acetylated lysine sites, sequence context of the acetylated lysine in the proteins was analyzed using the Motif-x program. These motifs exhibited different abundances. The KacG (16.6%, 395/2373), KacK (15.0%, 358/2373), KacH (10.5%, 250/2373), and KacT (10.4%, 247/2373) motifs were the most common ones (Figures 5A,B). The residues of alanine (F), histidine (H), lysine (K), asparagine (N), proline (P), threonine (T), and tyrosine (Y) were highly enriched at the +1 position near the Kac site, and alanine (A), aspartic acid (D), glycine (G), and tyrosine (Y) and H were more commonly observed at the −1 position, and isoleucine (I), G, H, enrichment were more commonly observed at the −2 position (Figure 5C).

To understand the locations of acetylated and/or non-acetylated lysine in the secondary structures of proteins (alpha-helix, beta-strand, and coil), the structural analysis of proteins containing lysine was performed. The results indicated that most acetylated sites were located at the beta-strand and coil and were surface-accessible (Figures 5D,E), but the acetylated lysine located in the alpha-helix region showed lower probability than non-acetylated lysine (Figure 5D).

### Cluster Analysis Based on Acetylation Levels of Proteins

**Figure 6:**
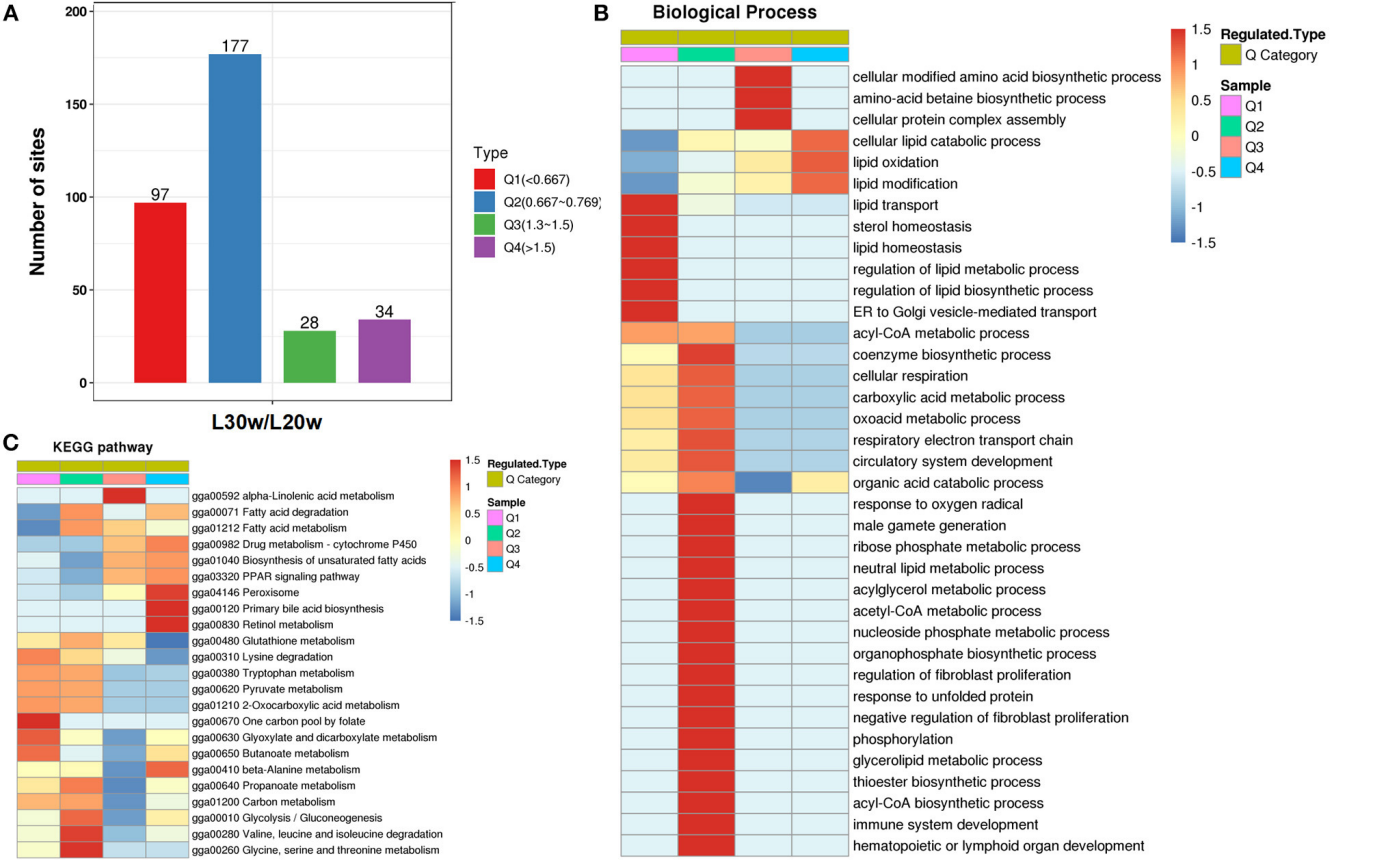
Cluster analysis of differential kac proteins based on their differential acetylation multiples. **(A)** Distribution of differentially acetylated proteins. The differentially acetylated proteins were classified into four groups according to their fold changes of acetylation levels: Q1 (0 < Ratio ≤ 1/1.5), Q2 (1/1.5 < Ratio ≤ 1/1.2), Q3 (1.2 < Ratio ≤ 1.5) and Q4 (Ratio > 1.5). **(B)** GO enrichment analysis of differential kac proteins in four clusters. Only the significantly enriched (*p* ≤ 0.05) biological process categories are shown. **(C)** KEGG enrichment analysis of differential kac proteins in four clusters. Only the significantly enriched (*p* ≤ 0.05) pathways are shown.

To investigate the protein function with the alteration levels of acetylation modification, the acetylated proteins were classed into four categories according to their differential acetylation multiples (Figure 6A and Supplementary Table 3). The proteins with highly significant downregulated acetylation levels in Q1 category were significantly enriched in lipid homeostasis-related biological functions, such as lipid transport (*p* < 0.01), and ER to Golgi vesicle-mediated transport (*p* = 0.03; Figure 6B). These proteins were enriched in the pathways of one carbon pool by folate (*p* < 0.01), such as methylenetetrahydrofolate dehydrogenase (Mthfd1) and 5-aminoimidazole-4-carboxamide ribonucleotide formyltransferase/IMP cyclohydrolase (Atic); pyruvate metabolism (*p* = 0.01), such as aldehyde dehydrogenase (Aldh) 9a1, dihydrolipoyl dehydrogenase (Dld), and Mdh2; and some amino acid metabolism, such as tryptophan metabolism (Figure 6C). The PwSDALs in Q2 category contained the enzymes related to cellular lipid catabolic process (*p* < 0.01), acyl-CoA metabolic process (*p* < 0.01), nucleotide catabolism, and cellular respiration (Figure 6B). The pathways significantly enriched in Q2 category were similar with Q1 category: pyruvate metabolism (*p* = 0.01), such as pyruvate dehydrogenase E1 subunit alpha 1 (Pdha1), Acac and Acss1l, and some amino acid metabolism, such as tryptophan metabolism (*p* < 0.01), also including fatty acid degradation (*p* = 0.01), such as acyl-CoA dehydrogenase short/branched chain (Acadsb), acyl-CoA dehydrogenase long chain (Acadl), glutaryl-CoA dehydrogenase (Gcdh), and Acaa2 and glycolysis/gluconeogenesis (*p* = 0.04), such as triosephosphate isomerase 1 (Tpi1; Figure 6C). In addition, PwSDALs were involved in xenobiotics by cytochrome P450, such as aldo–keto reductase family 7 member A2 (Akr7a2), microsomal glutathione S-transferase 1 (Mgst1), Gsta4l and Gstk1, and some amino acid metabolism (Supplementary Figure 4B). These results not only emphasized the key roles PwSDALs played in pyruvate metabolism and fatty acid degradation but also uncovered one carbon pool by folate pathway which were involved in hepatic metabolism.

In contrast to Q1 and Q2 categories, the PwSUALs in the Q4 category were mainly involved in the lipid catabolic process. These proteins were enriched in the pathways of peroxisome (*p* < 0.01), such as diazepam binding inhibitor (Dbi), sterol carrier protein 2 (Scp2) and catalase (Cat) and lipid oxidation (*p* = 0.01), such as enoyl-CoA isomerase (Eci) and enoyl-CoA hydratase and 3-hydroxyacyl CoA dehydrogenase (Ehhadh). Other proteins were involved in the TCA cycle, such as oxoglutarate dehydrogenase (OGDH); drug metabolism-cytochrome P450, such as glutathione S-transferase alpha 4 (Gsta4l) and glutathione S-transferase kappa 1 (Gstk1); and some amino acid metabolism, which would mainly regulate the metabolism of polyunsaturated fatty acid (PUFA) in peroxisome (Figures 6B,C). The PwSUALs in Q3 category were largely enriched in amino acid biosynthetic process and cellular protein complex assembly (*p* = 0.04) and significantly enriched in the pathways of alpha-linolenic acid metabolism (*p* < 0.01); fatty acid degradation (*p* = 0.03), such as acyl-coA oxidase (Acox1) and acyl-CoA acyltransferase (Acaa) 1, and PPAR signaling pathway (*p* = 0.01), such as protein Dbi (Figure 6C). In addition, the upregulation of acetylation in some glutathione transferases, such as Gsta4l, Gstk1, and deoxynucleotidyl transferase terminal interacting protein 2 (Dnttip2) linked acetylation to liver detoxification mechanism (Supplementary Figure 4A). Collectively, acetylation modifications of the enzymes involved in lipid homeostasis-related biological functions, fatty acid degradation, TCA cycle, cytochrome P450, and PPAR signaling pathway contributed to adapting to the changes of hepatic lipid metabolism during the egg-laying period.

### The Interaction Network of the Acetyl-Proteins

**Figure 7:**
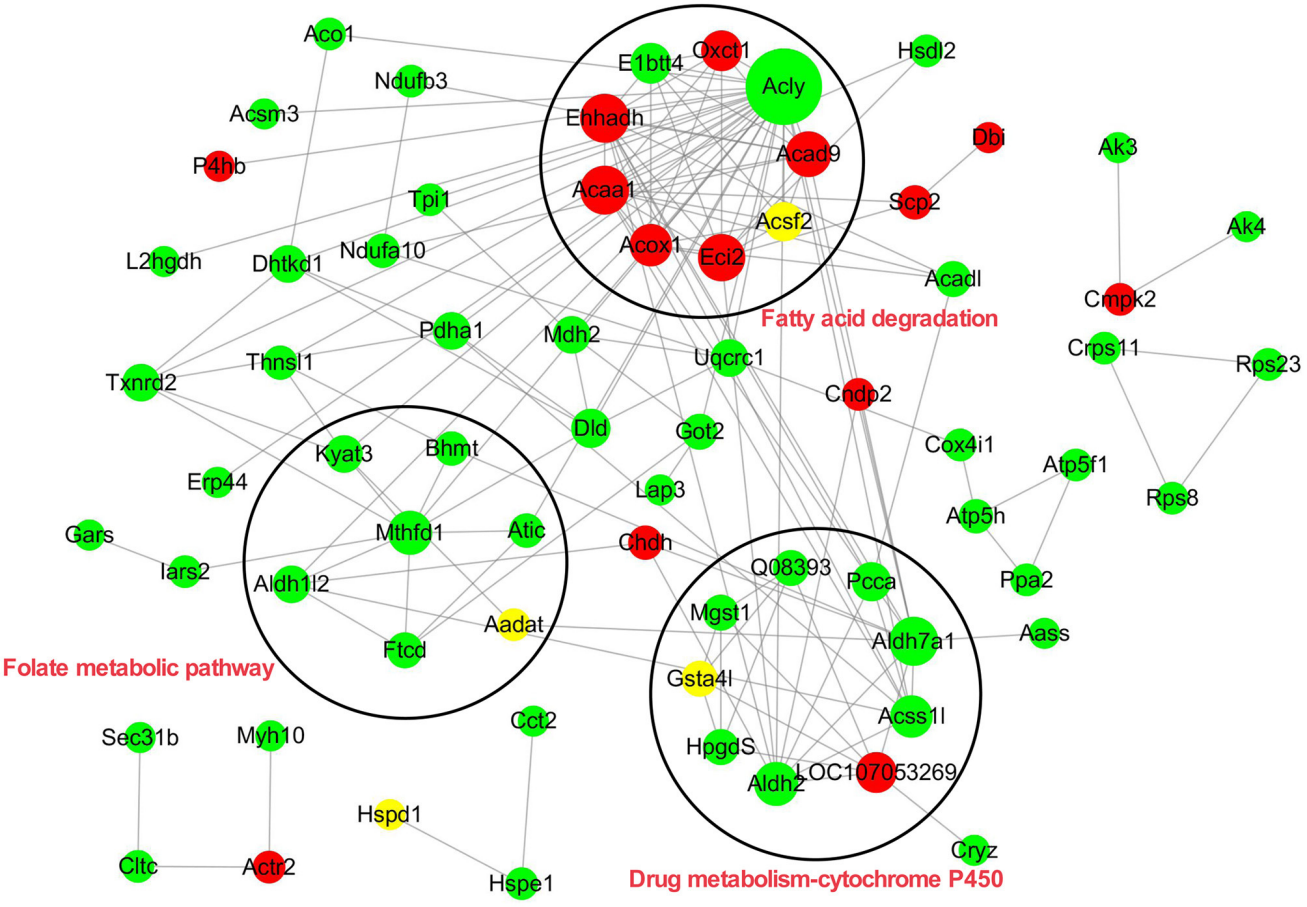
Protein–protein interaction network of all differential kac proteins.

To elucidate the interaction relationship among the acetylated proteins, the proteins with significantly differential acetylation levels were used to construct the protein–protein interaction (PPI) network. A total of 70 acetylated proteins were mapped to the protein interaction database by PPI analysis. Three clusters were displayed in the network. Cluster 1 was enriched with proteins involved in fatty acid degradation. Among these proteins, Acly, a key enzyme regulating lipid synthesis by converting cytosolic citrate to acyl-CoA, exhibited the highest degree of connectivity. In addition, there were five PwSUALs with high interaction degrees in the cluster, such as acyl-CoA dehydrogenase family member 9 (Acd9), Acaa1, Ehhadh, Eci2, and Acox1, enriched in this cluster, and involved in fatty acid degradation (Figure 7 and Table 2). Cluster 2 was enriched with proteins associated with drug metabolism-cytochrome P450 (Figure 7). There were three PwSDALs, including Aldh2, Aldh7a1, and Acss1l, and a PwSUAL LOC107053269 displayed high interaction degrees (Figure 7 and Table 2), while in cluster 3, four PwSDALs including formimidoyltransferase cyclodeaminase (Ftcd), Mthfd1, Atic, and Aldh1l2 were involved in folate metabolic pathway, which was involved in fatty acid degradation [(39); Figure 7].

**Table 2:** Top 20 hub-proteins in PPI network.

**Protein accession**	**Degree**	**Kac site (up/down/all)**	**Regulated type**	**Gene name**
F1P269	58	0/2/21	Down	ACLY
E1C4W4	24	0/1/7	Down	ALDH7A1
F1NB64	24	1/0/6	Up	ACAA1
E1C1T9	24	1/1/16	Up	EHHADH
E1BW06	22	3/0/12	Up	ECI2
F1NEF6	20	2/0/17	Up	ACAD9
E1BT93	18	0/3/16	Down	ALDH2
F1NMC3	18	0/1/9	Down	MTHFD1
F1NY37	16	5/0/8	Up	ACOX1
E1BZT9	16	2/0/4	Down	ACSS1L
F1NTZ0	14	3/0/7	Up	LOC107053269
F1N9Z7	14	1/0/1	Up	OXCT1
E1BTT4	14	0/4/10	Down	E1BTT4
Q5ZM32	12	0/9/17	Down	DLD
F1P0M2	12	0/2/11	Down	PCCA
E1BS15	12	1/1/11	Mix	ACSF2
F1NQC6	10	0/1/4	Down	DHTKD1
E1BVT3	10	0/2/12	Down	MDH2
E1C934	10	0/2/2	Down	KYAT3

### Integrated Analysis of Proteome and Acetyl-Proteome

**Figure 8:**
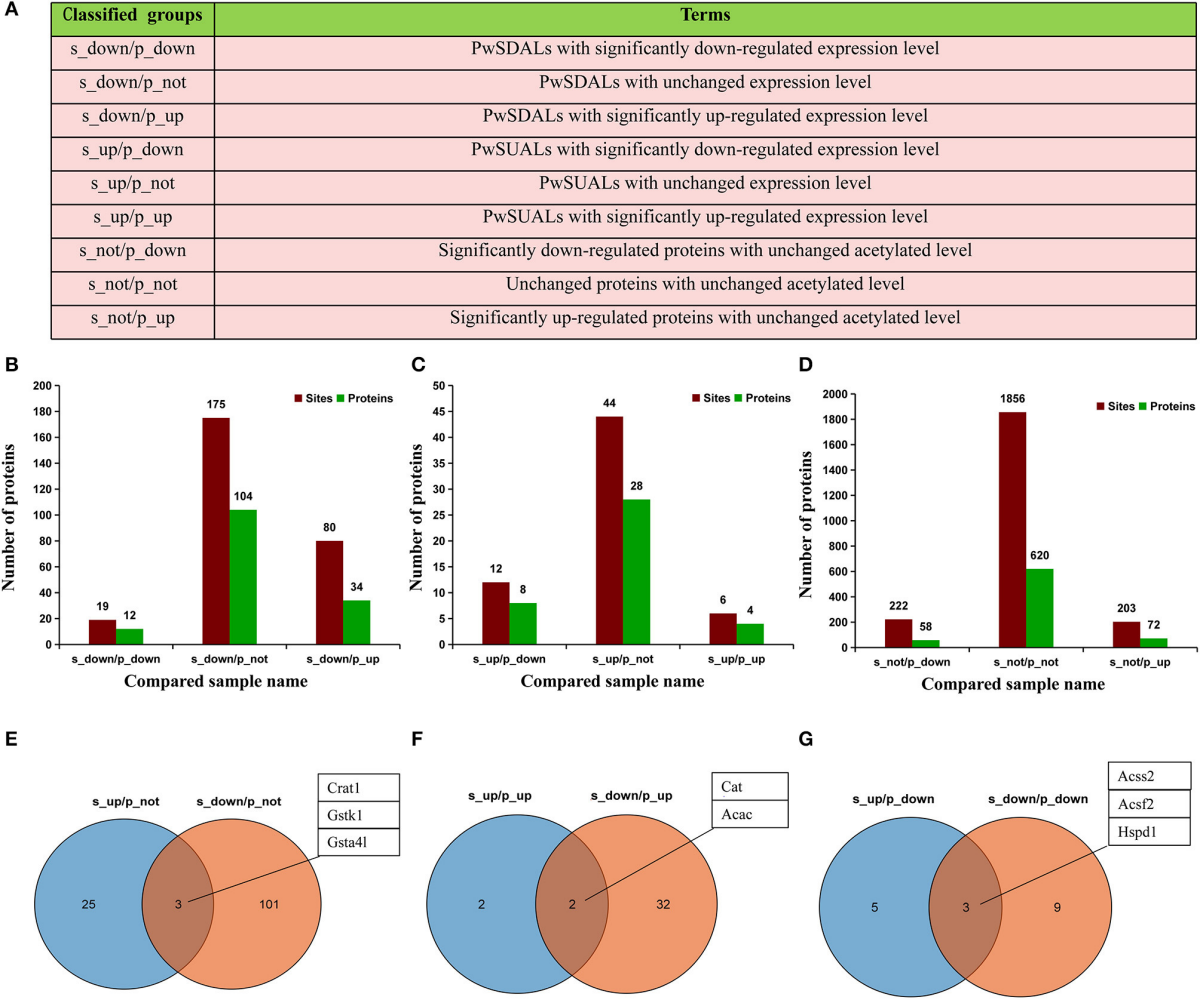
Integrated analysis of proteome and acetyl-proteome data. **(A)** The proteins were divided into nine groups according to both their expression levels and acetylation levels. **(B–D)** The numbers of Kac sites and corresponding proteins in each group. Dark green represents the number of proteins, and dark red represents the number of Kac sites in the same group. **(B)** Three groups including PwSDALs with unchanged expression level (104/150), PwSDALs with downregulated expression level (12/150), and PwSDALs with upregulated expression level (34/150). **(C)** Three groups including PwSUALs with downregulated expression level (8/40), PwSUALs with unchanged expression level (28/40), PwSUALs with upregulated expression level (4/40). **(D)** The 782 proteins for which the differences of their acetylation levels between pre- and peak-laying hens did not reach the threshold value, including upregulated proteins (58/782), unchanged proteins (620/782), and downregulated proteins (72/782). **(E–G)** The Venn diagrams show the proteins which showed both significantly up- and downregulated acetylation levels. **(E)** Three PwSU_DALs with unchanged protein levels. **(F)** Two PwSU_DALs with upregulated protein levels. **(G)** Two PwSU_DALs with downregulated protein levels.

To further understand the epigenetic effect of protein acetylation on liver metabolism of hens from the pre-laying to peak-laying physiological stages, the integrated analysis of proteome and acetylated proteome was performed. Firstly, the proteins were classified into nine groups according to their expression levels and acetylation levels (Figure 8A and Supplementary Tables 4, 5). Subsequently, pathway analysis was performed based on the background of 1,313 acetylated proteins (Figure 8).

**Figure 9:**
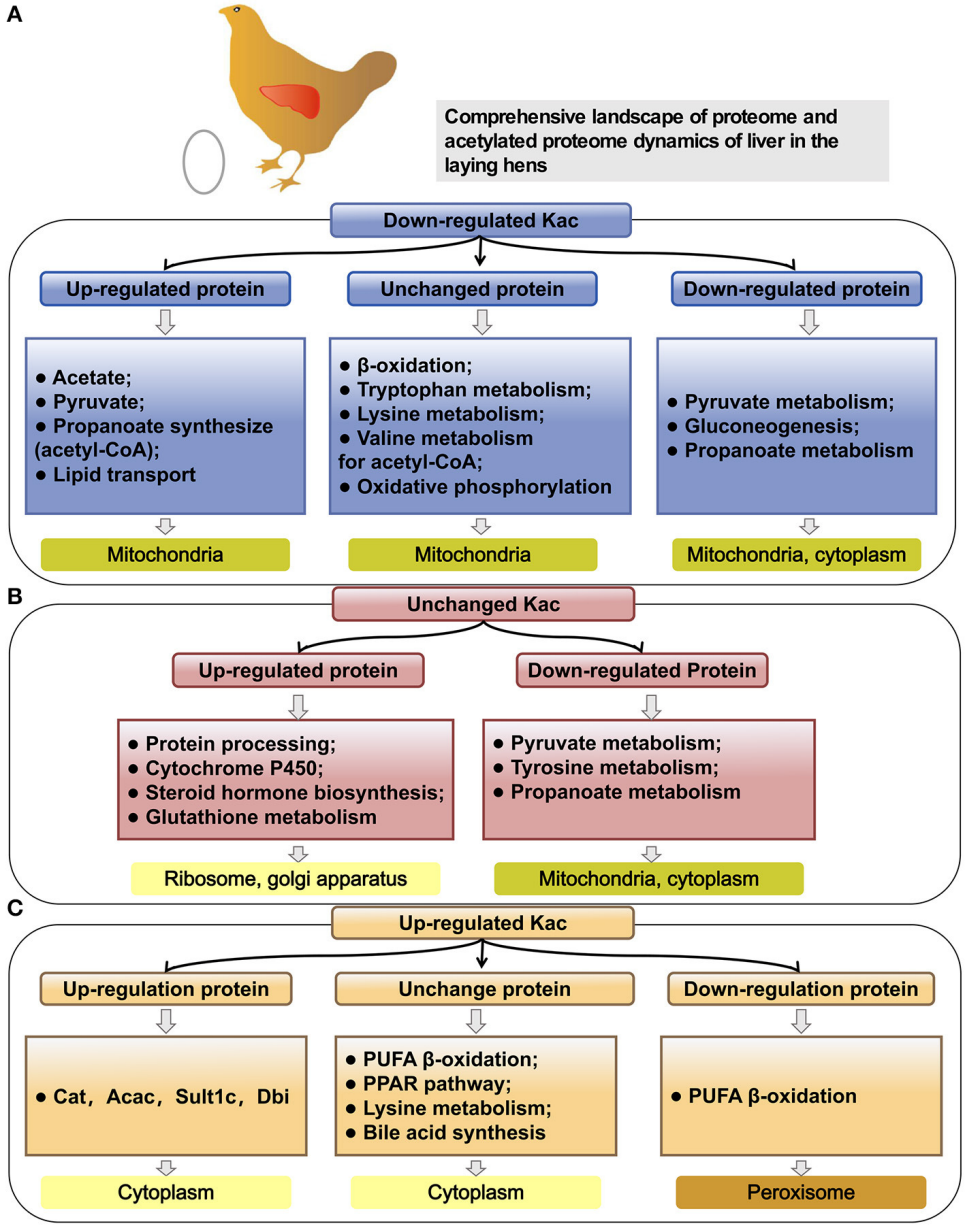
Pathway analysis of different protein groups based on the same background of 1,131 kac proteins. **(A)** Pathway analysis of PwSDALs with unchanged expression level (104/150), downregulated expression level (12/150) and upregulated expression level (34/150). **(B)** Pathway analysis of PwSUALs with downregulated expression level (8/40), unchanged expression level (28/40), and upregulated expression level (4/40). **(C)** Pathway analysis of two groups based on the differences of their acetylation levels in livers between pre- and peak-laying hens, including upregulated proteins (58/782) and downregulated proteins (72/782).

Among the 150 PwSDALs, 104 proteins with unchanged expression level were significantly enriched in the pathways of fatty acid degradation (*p* < 0.01), such as proteins Acadsb, Gcdh, Hadha, Acadl, Hadh, Aldh7a1, and Acaa2, and oxidative phosphorylation (*p* = 0.04), such as proteins inorganic pyrophosphatase 2 (Ppa2), NADH: ubiquinone oxidoreductase subunit A10 (Ndufa10), and ATP synthase F1 subunit alpha pseudogene 2 (Atp5f1) in mitochondria and cytoplasm (Figures 8B, 9). There were 12 downregulated expression proteins, and only one protein, Acss1l, was significantly enriched in the pathways of pyruvate metabolism (*p* = 0.02) and gluconeogenesis (*p* = 0.03; Figures 8B, 9). The 34 upregulated expression proteins were involved in the TCA cycle, such as protein Idh2; biosynthesis of unsaturated fatty acids, such as acyl-CoA thioesterase 1-like (Acot1l) and Fads2; and some amino acid metabolism processes (Figures 8B, 9). These results indicated that deacetylation modifications were closely associated with fatty acid oxidation and oxidative phosphorylation, and the alteration of protein abundance and acetylation level together affected the aerobic oxidation of sugar in peak-laying hens.

For the 40 PwSUALs, 28 proteins with unchanged expression level were significantly enriched in the PUFA oxidation pathways in peroxisome (*p* < 0.01), such as proteins Acox1, Ehhadh, Scp2, and Eci2; primary bile acid synthesis (*p* = 0.02), such as Scp2 and Hsd17b4; and lysine metabolism (*p* = 0.02), such as proteins Ehhadh and Acaa1 (Figures 8C, 9). Eight downregulated proteins were associated with fatty acid degradation and glutathione pathway, such as Dnttip2 (Figures 8C, 9). The remaining four proteins were upregulated in their protein expression levels. Among them, sulfotransferase family 1C member 2 (Sult1c) regulated steroid hormone sulfur transfer, and Dbi had the ability to bind long-chain acyl-CoA esters, and then regulated the lipid metabolism (Figure 9). These results demonstrated that the core role of hyperacetylation of lysine was to affect PUFA oxidation via acetylation of Acox1, Ehhadh, Scp2, and Eci2 in peroxisome.

A total of 750 proteins showed no significant difference in the acetylation levels in livers of peak-laying hens compared with the pre-laying hens (1/1.5 ≤ L30w/L20w ratio ≤ 1.3 and *p* ≥ 0.05). Among those, 72 proteins with increased expression level affected the protein translation and processing (*p* < 0.01), such as ribosome complex-related proteins (Figures 8D, 9). The 58 proteins with downregulated expression level were significantly enriched in the pathways of pyruvate metabolism (*p* < 0.01), glycolysis/gluconeogenesis (*p* = 0.04), and some amino acid metabolism (Figures 8D, 9). There were also 620 proteins with unchanged expression levels that contained unchanged abundance of Kac sites (Figures 8D, 9). These results indicated that, in addition to the difference in fatty acid degradation, the energy metabolism in the liver was also stronger in 30 w birds than that in 20 w birds.

Notably, some proteins could be simultaneously classified as with upregulated acetylation levels and downregulated acetylation levels because they underwent acetylation in some lysine sites and deacetylation in other lysine sites. A similar mechanism of protein acetylation modifications also appeared in liver of peak-laying hens compared to that in pre-laying hens. These proteins were extracted from the above nine groups. There were three proteins which were unchanged in their expression levels but simultaneously possessed significantly upregulated and downregulated acetylation sites (PwSU_DALs), including acyl-CoA transport-related protein carnitine O-acetyltransferase (Crat1), Gstk1, and Gsta4l (Figure 8E). In addition, there were two PwSU_DALs with upregulated expression levels, including Cat and Acac, and the latter is a rate-limiting enzyme involved in fatty acid synthesis (Figure 8F). There were three PwSU_DALs with downregulated expression levels, including Acss2, acyl-CoA synthetase family member 2 (Acsf2), and 60 kDa heat shock protein (Hspd1; Figure 8G). It is worth noting that Acss2 is not only an important enzyme that participates in the production of acyl-CoA via pyruvate metabolism but also associated with fatty acid degradation via drug metabolism–cytochrome P450. According to these results, it was clear that PwSU_DALs were key enzymes in glutathione metabolism, acyl-CoA, and fatty acid synthesis. These proteins were simultaneously acetylated and deacetylated at different lysine sites and regulated protein activities, reflecting a more complex mechanism of acetylation modification of proteins involved in liver detoxification and fatty acid metabolism.

## Discussion

**Figure 10:**
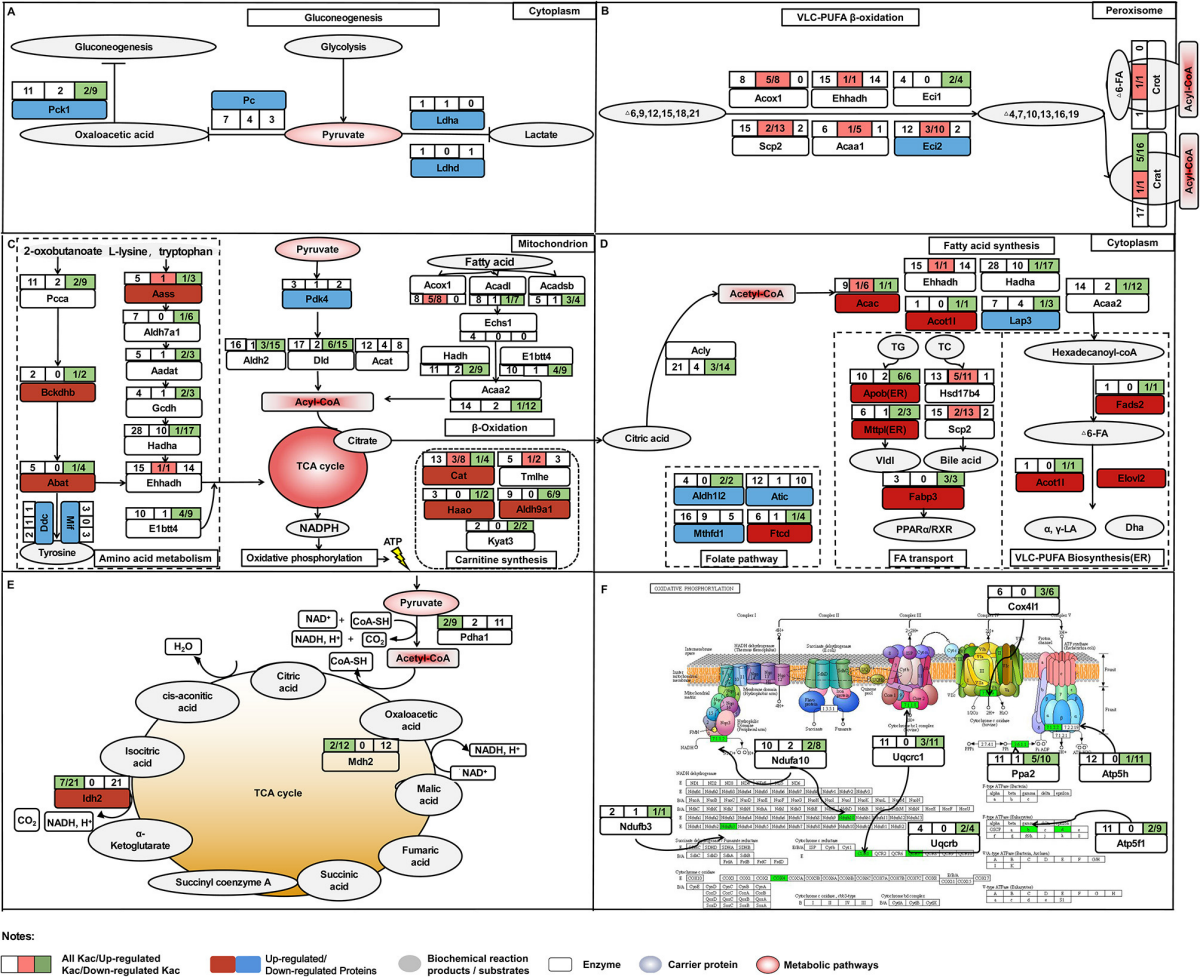
The comprehensive proteome and acetyl-proteome atlases of liver in pre-laying and peak-laying hens. The acetylated proteins involved in the aerobic oxidation process of sugar including glycolysis, pyruvate metabolism, TCA cycle and oxidative phosphorylation, fatty acid b-oxidation, carnitine synthesis, and some amino acid metabolism. **(A)** The acetylated proteins involved in glycolysis. **(B)** The acetylated proteins involved in pathways of PUFA oxidation in peroxisome. **(C,D)** The acetylated proteins involved in pathways including fatty acid synthesis, polyunsaturated fatty acid biosynthesis, fatty acid transport, and folate pathway in cytoplasm. **(E)** The acetylated proteins involved in TCA cycle. **(F)** The acetylated proteins involved in the process of oxidative phosphorylation. Dark red boxes represent significantly upregulated proteins, light blue boxes represent significantly downregulated proteins. The circle indicates the processes of TCA cycle. Three consecutive squares represent the number of Kac sites (all/up/down). Top left box shows the numbers of all Kac sites for an individual protein. Top right box shows the numbers of all upregulated Kac sites for an individual protein; the number before the semicolon represents the number of significantly upregulated Kac sites. Bottom right box indicates the number of all downregulated Kac sites for an individual protein; the number before the semicolon represents the number of significantly downregulated Kac sites. The pink color represents the protein with significantly upregulated Kac sites, while green color represents significantly downregulated Kac sites.

In hens, with reaching the peak-laying period, the liver undergoes a serial of great physiological changes to meet the needs for egg-laying. The most notable ones are the enhanced lipid metabolism in both catabolic and anabolic directions in liver (40) and the activated protein synthesis and energy metabolism (35). However, the detailed regulatory mechanisms regarding the adaption of the physiological changes from pre-laying to peak-laying stage in liver of hens remain unknown. In the present study, for the first time, the global atlas of liver proteome and acetylated proteome were conducted to annotate the functional proteins and epigenetic effects of lysine acetylation on liver metabolism at the egg-laying stage. Compared to juveniles, most of the upregulated proteins in peak-laying are involved in fatty acid synthesis, fatty acid transport, unsaturated fatty acids synthesis, cholesterol synthesis, TG synthesis, and steroid hormone synthesis processes. The mostly upregulated proteins were Vtg, Fabp3, Dhcr24, Sqle, Fads1, and Elovl2 (Figure 10B), as confirmed by our previous transcriptome studies in liver of chicken (32, 41, 42). Meanwhile, as expected, some of the upregulated proteins in peak-laying hens were involved in protein synthesis (such as the members of the Rpl family; Supplementary Figure 2) and energy metabolism (such as the members of cytochrome *c* oxidase; Figure 10F), as confirmed by previous transcriptome profiling of the liver among the juvenile and laying stages in chickens (43, 44).

Reversible acylation modifications provide an extremely efficient mechanism for the management of protein function (45). Lysine acetylation levels were determined by the combined actions of lysine acetyltransferases (KATs) and deacetylases (KDACs), both of which may select their substrates with sequence preference of Kac sites (46, 47). Our proteome profiling detected many KATs and KDACs, such as the N-acetyltransferase (NAT) gene family and Sirt gene family, but only dihydrolipoamide S-acetyltransferase (DLAT) was significantly downregulated in liver of 30 w birds, while abundance of the others showed no significant difference in livers between pre- and peak-laying birds. It implied that the KATs and KDACs could alter biological functions of certain proteins to adapt the physiological changes by selectively modifying different substrates and acetylated sites of the proteins. In addition, we found that (G)K, K(H), and K(Y) were the favorable Kac sites, which are confirmed by other related acetyl-proteome studies on liver (27, 48, 49). Further analysis of the secondary structure of the protein between acetylated and unacetylated regions revealed that, in agreement with previous findings, Kac sites were usually located in the conservatively ordered secondary structure (49), which might result in a greater chromatin opening, thus facilitating transcriptional progression (50).

Increasing evidence suggested that most enzymes associated with glucose and fatty acid metabolism were acetylated at critical lysine sites in liver, which played significant roles in controlling energy metabolism (22). In the current study, proteins with the alteration levels of acetylation modification showed a significant correlation with fatty acid degradation. The PwSUALs with unchanged expression level, such as Acox1, Ehhadh, and Scp2, were related to PUFA oxidation in peroxisome (Figure 10C), while the PwSDALs with unchanged expression level, such as Acadl, Hsdh, and Acaa2, were related to medium and short FA oxidation in mitochondria (Figure 10A). A previous study found that hyperacetylation of Ehhadh in different lysine residues could increase its enzymatic activity and activate the FA oxidation (51). Therefore, hyperacetylation of Ehhadh might positively regulate PUFA oxidation in chicken liver. It was indicated that fructose-supplemented HFD led to increased acetylation of Acadl, which was associated with decreased fat metabolism (52). These results suggested that an increase of mitochondrial fatty acid oxidation was achieved by promoting mitochondrial fatty acid oxidation via deacetylating Acadl, Hadh, and Acaa2 in the liver of laying hens. Additionally, acetylation modification of proteins activated fatty acid oxidation to yield acyl-CoA, which could be used for the fuel of TG and cholesterol production. In addition, four PwSDALs including Mthfd1, Atic, Ftcd, and Aldh1l2 were significantly enriched in one carbon pool by folate pathway, which had been shown to be able to regulate hepatic lipid metabolism via a PPAR-alpha-regulated lipid catabolic pathway (39). It had been pointed out that Mthfd1 (53), Atic (54), and Aldh1l2 (55) involved fatty acid degradation. Therefore, acetylation might be involved in the folate pathway to participate in fatty acid degradation in chicken liver. Other differentially acetylated proteins were notably enriched in fatty acid elongation, biosynthesis of unsaturated fatty acids, peroxisome, primary bile acid biosynthesis, PPAR signaling pathway, and drug metabolism-cytochrome P450, which played important roles in live lipid metabolism of chicken (41). It was thus clear that fatty acid degradation pathway was also activated to provide adequate substrates satisfying lipid synthesis and energy metabolism in liver of peak-laying hens.

Besides lipid metabolism, active energy metabolism is required for the demands of egg production via aerobic oxidation of sugar that is followed by glycolysis, pyruvate metabolism, TCA cycle, and oxidative phosphorylation in liver of peak-laying hens. Multiple alternative fluxes were available for metabolism of pyruvate in the liver including (i) gluconeogenesis *via* Pc, (ii) decarboxylation to acyl-CoA via PDH complex, (iii) transamination to alanine via alanine transaminase, or reduction to lactate *via* lactate dehydrogenase (56). In this study, pyruvate metabolism was the one of the most representative processes enriched in the downregulated proteins. A decreased protein level of Pc inhibited pyruvate toward gluconeogenesis (57). Consistently, our data showed that the protein levels of Pc and Pck1, two rate-limiting enzymes of the gluconeogenesis, were significantly decreased in livers of peak-laying hens, suggesting that pyruvate production to be used toward gluconeogenesis was inhibited *via* Pc. The Ldha was a central functional module in the pathway of lactate synthesis (58, 59), and its abundance was significantly decreased in liver of peak-laying hens compared with that in pre-laying hens, indicating that pyruvate was decreasingly converted to lactate. Moreover, the expression of Pdk4 was significantly decreased with the advance of laying peak. Previous study indicated that decreased protein level of Pdk4 dephosphorylated PDH complex activated the PDH complex for conversion of pyruvate to acyl-CoA (38). In addition, the Pdha1 is a core subunit of PDH complex. It was reported that deacetylated Pdha1 increased the activity of PDH (60, 61). In the present study, the acetylation level of Pdha1 protein was significantly downregulated, suggesting that the reaction of pyruvate into acyl-CoA was activated by promoting the catalytic activity of PDH via deacetylation of Pdha1 in the peak-laying stage (Figure 10A). Clearly, the activated metabolic pathway leading to conversion from pyruvate to acyl-CoA was another alternative to meet the necessary prerequisite, which was acyl-CoA accumulation, for fatty acid synthesis and energy metabolism in liver of peak-laying birds.

Furthermore, Pdha1, Idh2, and Mdh2 are three key proteins which elevate the reduction of NADP+ to NADPH for oxidative phosphorylation via TCA cycle (Figures 10D,E). Previous studies indicated that catalytic activities of deacetylated Pdha1 (60), Idh2 (62), and Mdh2 (63) were increased. Meanwhile, several other studies had confirmed that deacetylated proteins involved in oxidative phosphorylation activate complex of electron respiratory chain for conversation of NADPH to ATP (64–66). Consistently, in our study, the acetylation levels of proteins involved in oxidative phosphorylation were down regulated with unchanged expression levels, implying that the deacetylated proteins are involved in the activation of TCA cycle and oxidative phosphorylation, which provide energy requirements for laying hens. These results suggested that the activated energy metabolism was closely related to the expression of pyruvate metabolism-related proteins and acetylation of oxidative phosphorylation-related enzymes in the liver of peak-laying hens.

Acetylated proteins play an important role in controlling mitochondrial function through interacting with each other. Interestingly, three clusters were identified in this PPI network, which included fatty acid degradation, folate pathway, and drug metabolism–cytochrome P450. Although cytochrome P450 does not intervene in the degradation of fatty acids in the β-oxidation cycle itself, previous studies have speculated an involvement of cytochrome P450 monooxygenases with decomposition of very-long-chain fatty acids and branched-chain fatty acids (67). Among proteins in the networks, deacetylated Acss1l and acetylated Acaa1, Ehhadh, Eci2, Acad9, and Acox1 were key enzymes regulating fatty acid degradation in laying hens. In addition, there were two proteins, Gsta4l and Gstk1, enriched in drug metabolism–cytochrome P450. They were two critical enzymes responsible for the detoxification of reactive lipid aldehydes (68). To detoxify, mice adapted to a higher expression level of glutathione S-transferase as a protective mechanism to eliminate increased reactive oxygen species [ROS; (69)]. In chicken, active energy metabolism and lipid metabolism may cause toxicity via lipid peroxidation, as well as activating detoxificated enzymes in the liver of peak-laying hens (70). However, PwSU_DALs including Gsta4l and Gstk1 with unchanged expression levels were found in liver, suggesting that acetylation of the proteins was involved in liver detoxification.

To further understand the effects of the post-translational acetylation on hepatic lipid metabolism, we performed integrated analysis of proteome and acetylated proteome. We found that the fatty acid degradation and energy metabolism were mainly regulated via acetylation, but the other metabolic pathways were influenced by both protein expression and acetylation modification. Some key liver metabolism-related proteins showed inconsistent trends in protein and acetylation abundance, which was involved in pyruvate metabolism and gluconeogenesis (such as Acss1l), fatty acid desaturases (such as Fads2), peroxisomal beta-oxidation (such as Acot1l), lipid transport (such as Scp2), and glutathione pathway (such as Dnttip2). As mentioned above, deacetylation of Acss1 enhanced its activity. In addition, PwSU_DALs Crat1 was involved in acyl-CoA transfer (71); Cat and Hspd1 was involved in regulation of mitochondrial ROS (52); and Acac, Acsf2, and Acss2 were involved in fatty acid synthase (1, 34). The effects of acetylation modifications on these proteins have been poorly studied. Previous studies had confirmed that the deactivation of Cat and Acss2 could be reactivated by the Sirt deacetylases (72, 73). Moreover, the effects of acetylation modification on the enzyme activity of PwSU_DALs were particularly complex because each acetylated lysine site might play its own regulatory role independently. For example, acetylation of P53 at K120 and K382 could activate the enzyme's activity and diminish P53-mediated apoptosis (74, 75), while acetylation of P53 at K317 negatively regulates P53 apoptotic activities with DNA damage (76). Our acetyl-proteome analysis showed that the acetylation levels of proteins were dynamically and intricately changed in the livers of pre- and peak-laying hens. This study provided a novel insight into the regulatory mechanism elucidating hepatic lipid metabolism in chicken, although the effects of acetylation modification on liver metabolism need to be further studied experimentally.

In conclusion, this study, for the first time, provides comprehensive proteome and acetyl-proteome atlases of liver in pre- and peak-laying hens (Figure 10). With the arrival of the peak-laying period, the upregulated proteins, such as Vtg, Fabp3, Dhcr24, Sqle, Fads1, and Elovl2, were used to fulfill the requirements of intense syntheses of proteins, lipids, and steroid hormones. The downregulated proteins, such as Pc, Pck1, and Ldha, were employed to inhibit gluconeogenesis and lactate synthesis. Meanwhile, downregulated Pdk4 and deacetylated Pdha1 could elevate the dephosphorylation and deacetylation of PDH complex, then regulate pyruvate flux by catalyzing the pyruvate-to-acyl-CoA conversion. In addition, the PwSUALs and PwSDALs with unchanged expression levels were involved in PUFA oxidation in peroxisome and medium and short FA oxidation in mitochondria, respectively. The activated fatty acid oxidation and pyruvate metabolism provided an alternate source of acyl-CoA. The accumulation of acyl-CoA, on the one hand, could be used for TCA cycle and subsequent oxidative phosphorylation for energy metabolism via PwSDALs and, on the other hand, could improve lipid synthesis to meet the needs of egg-laying (Figure 10). Other differentially acetylated proteins were notably involved in fatty acid elongation, biosynthesis of unsaturated fatty acids and primary bile acids, and detoxification. Overall, the above aspects worked together to promote hepatic lipid metabolism to adapt to the physiological changes from pre-laying to peak-laying stage in hens. This study provides a new insight into regulatory mechanism of the hepatic lipid metabolism in laying hens.

## Data Availability Statement

The datasets presented in this study can be found in online repositories. The names of the repository/repositories and accession number(s) can be found at: LC-MS/MS have been deposited at ProteomeXchange with identifier PXD024718.

## Ethics Statement

The animal study was reviewed and approved by the Institutional Animal Care and Use Committee (IACUC) of Henan Agricultural University, Zhengzhou, P.R. China.

## Author Contributions

ZW performed the majority of conceptualization, statistical analyses, and drafted the manuscript. DW and KJ performed protein extraction and western blotting and helped to prepare the manuscript. YG and ZL helped to manage the experimental animals and collect the samples. RJ and RH participated in the data analysis. GL and YT participated in the database search and functional enrichment analysis. HL participated in the design of the study and helped revise the manuscript. XK participated in the design of the study and critical discussion of results. XL conceived the study and provided overall supervision. All authors read and approved the final manuscript.

## Funding

This research was funded by the Key Project of NSFC-Henan Province Joint Fund (U1704233), Scientific Studio of Zhongyuan Scholars (NO. 30601985), Zhongyuan Science and Technology Innovation Leading Scientist Project (214200510003), and Program for Innovative Research Team in Science and Technology in University of Henan Province (21IRTSTHN022). Sequencing services were provided by Jingjie PTM BioLab (Hangzhou) Co. Ltd., Hangzhou 310018, China.

## Conflict of Interest

The authors declare that the research was conducted in the absence of any commercial or financial relationships that could be construed as a potential conflict of interest.

## Publisher's Note

All claims expressed in this article are solely those of the authors and do not necessarily represent those of their affiliated organizations, or those of the publisher, the editors and the reviewers. Any product that may be evaluated in this article, or claim that may be made by its manufacturer, is not guaranteed or endorsed by the publisher.
