# Supplementary material for: A Comprehensive Proteome and Acetyl-Proteome Atlas Reveals Molecular Mechanisms Adapting to the Physiological Changes From Pre-laying to Peak-Laying Stage in Liver of Hens (Gallus gallus)
Source: Front Vet Sci. 2021 Oct 21;8:700669. doi: 10.3389/fvets.2021.700669 (PMC8566343; doi:10.3389/fvets.2021.700669)

Supplementary Tables

Supplementary Table 1. Statistical information of differentially expressed proteins.

Supplementary Table 2. Statistical information of PwSDALs.

Supplementary Table 3. Hierarchical clustering information of PwSDALs.

Supplementary Table 4. The list of integrated analysis of proteome and acetyl-proteome datas.

Supplementary Table 5. Details of each group of proteins based on the integrated analysis of proteome and acetyl-proteome datas.

Supplementary Figures

Supplementary Figure 1. Study design and data quality of proteome. (A) The whole experimental work-flow for the study. **(B)** PCA analysis. **(C)** Hierarchical cluster analysis. **(D)** Elative standard deviation (RSD) of protein quantification.

Supplementary Figure 2. KEGG pathway analysis indicated that many up-regulated proteins were involved in the pathways of translation.

Supplementary Figure 3. Data quality of acetyl-proteome. (A) PCA analysis. **(B)** Elative standard deviation (RSD) of protein quantification. **(C)** The length of the peptide segments.

Supplementary Figure 4. GO and KEGG enrichment of differentially acetylated proteins. (A-B) GO enrichment analysis of up-regulated and down-regulated kac proteins in the liver of laying hens, respectively. Only the significantly enriched ($P \leq 0.05$) biological process, cellular component, and molecular function categories are shown. **(C-D)** KEGG enrichment analysis of up-regulated and down-regulated kac proteins in the liver proteome of laying hens, respectively. Only the significantly enriched ($P \leq 0.05$) pathways are shown.

Supplementary Figure 1

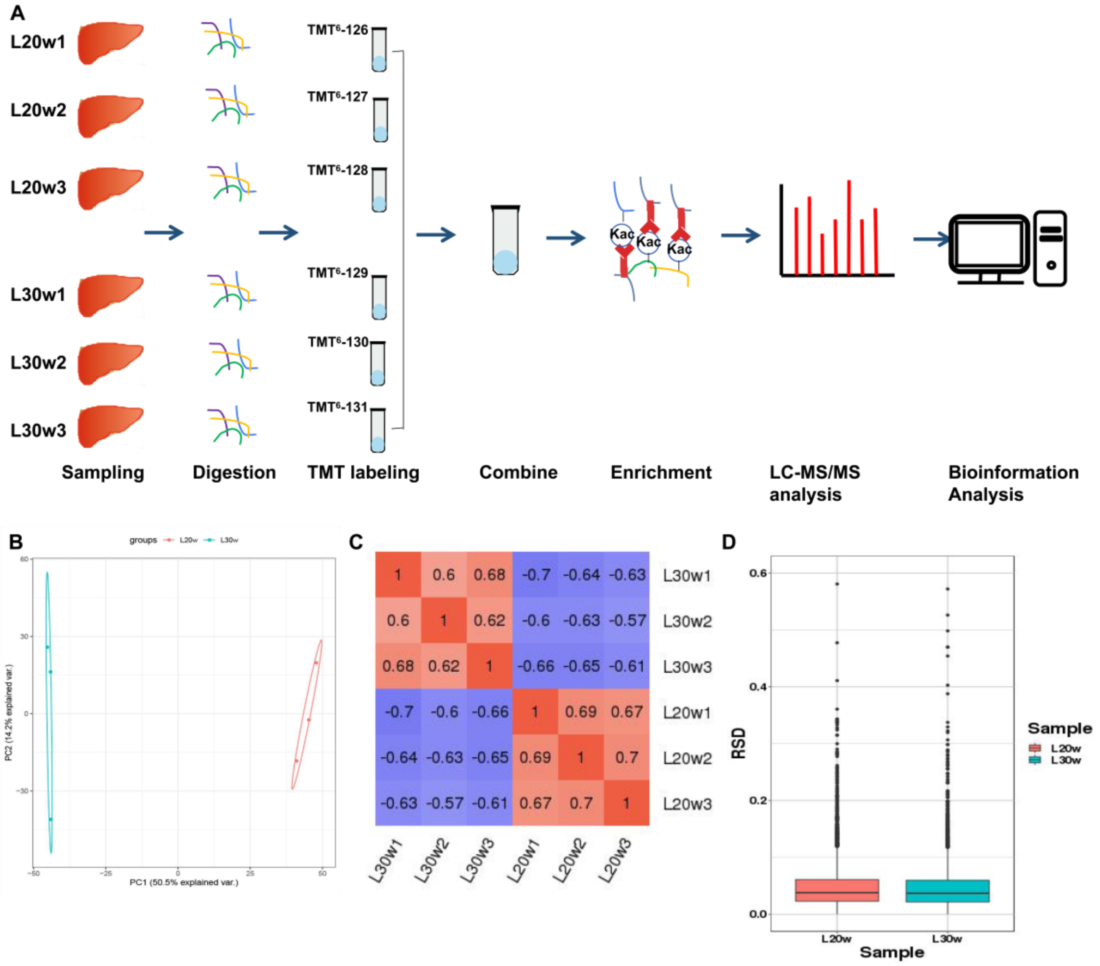

Supplementary Figure 2

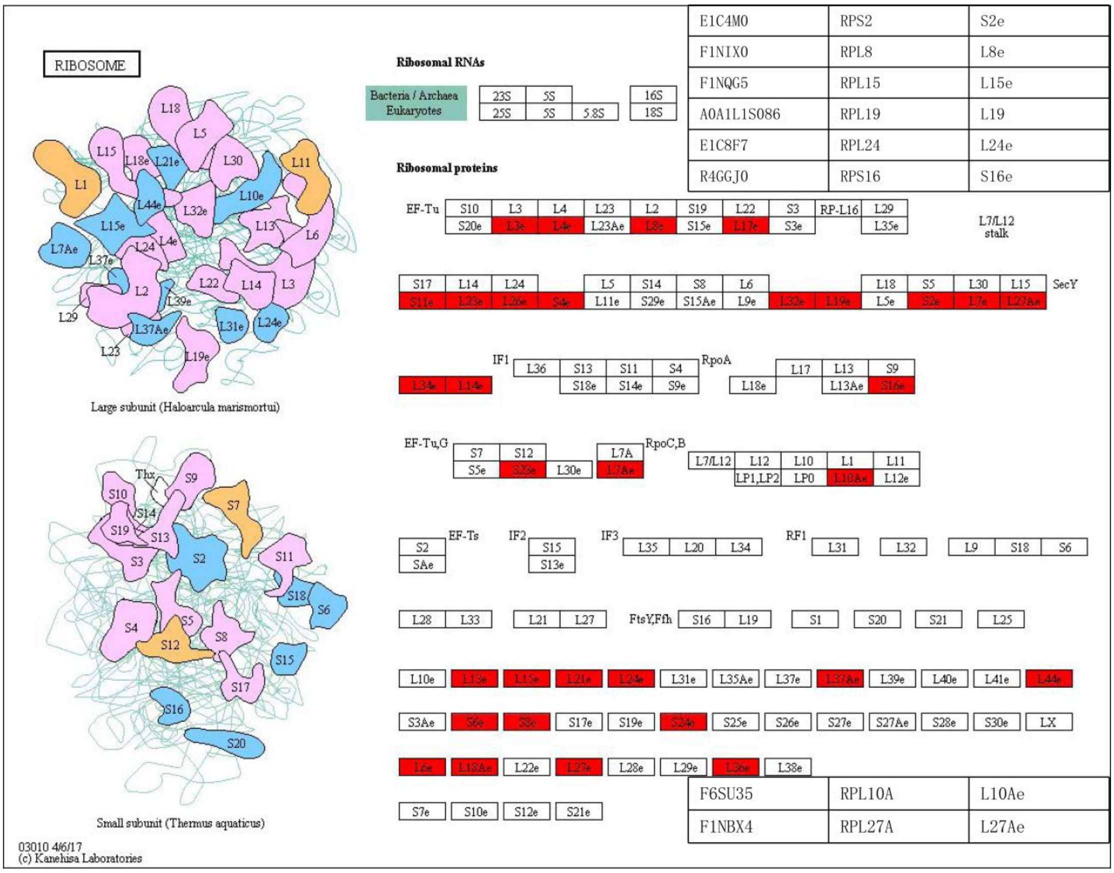

Supplementary Figure 3

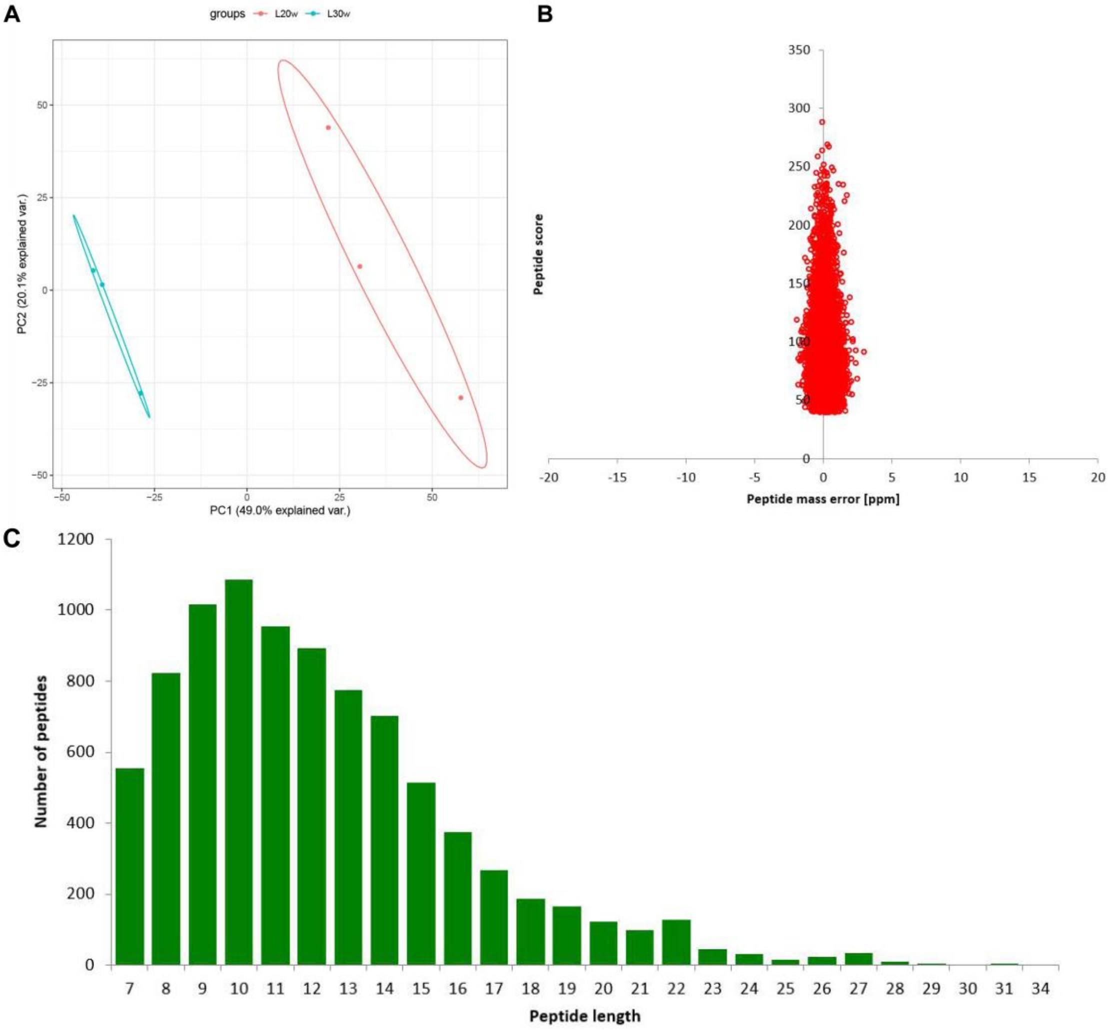

Supplementary Figure 4

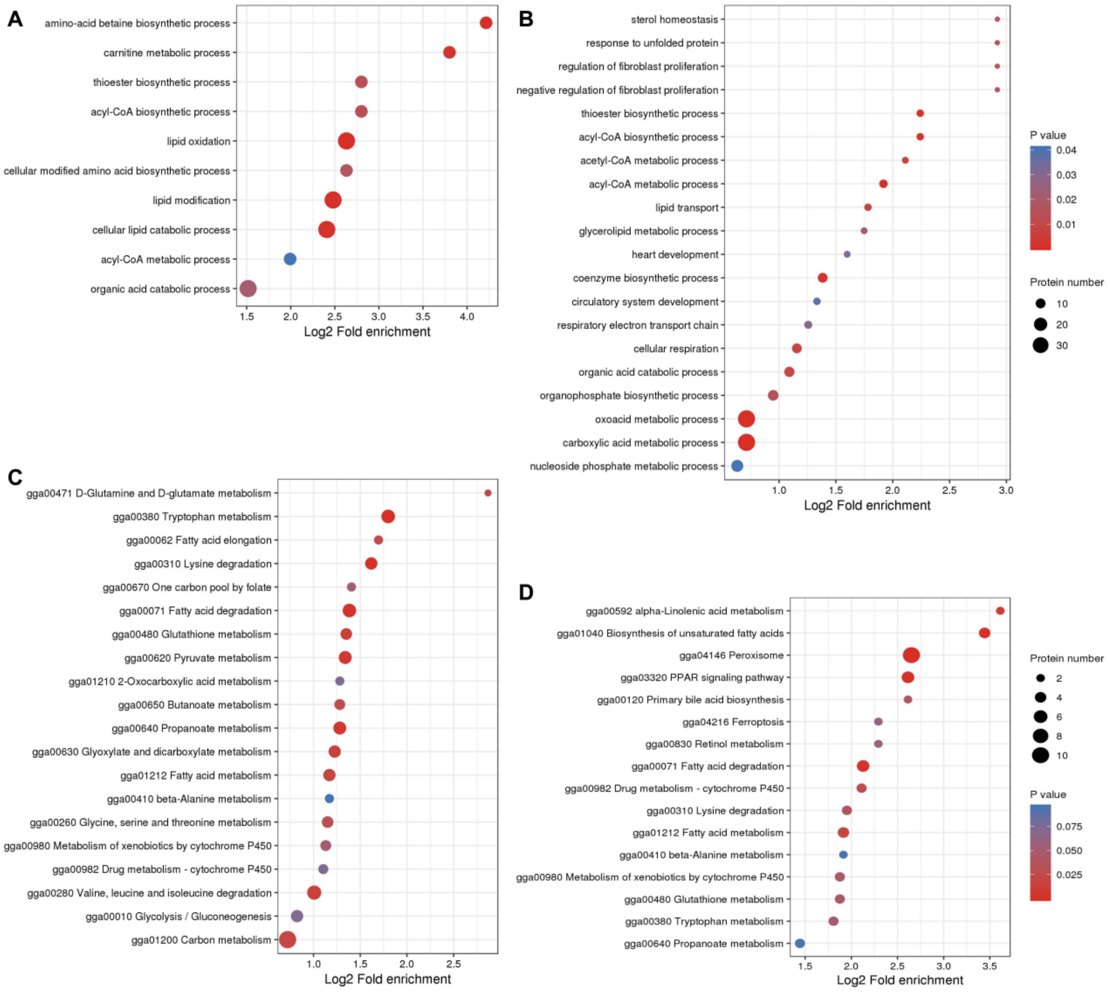

Supplement: Supplementary file 6 [file Data_Sheet_1.pdf]
